# Scaling of vagus nerve stimulation parameters does not achieve equivalent nerve responses across species

**DOI:** 10.1186/s42234-025-00174-9

**Published:** 2025-05-16

**Authors:** Eric D. Musselman, Ishani Raha, Nicole A. Pelot, Warren M. Grill

**Affiliations:** 1 Department of Biomedical Engineering, Duke University, Durham, NC USA; 2 Department of Electrical and Computer Engineering, Duke University, Durham, NC USA; 3 Department of Neurobiology, Duke University, Durham, NC USA; 4 Department of Neurosurgery, Duke University, Durham, NC USA

**Keywords:** Vagus nerve stimulation, Neural computational modeling, Peripheral nerve stimulation, Translation

## Abstract

**Background:**

Previous efforts to translate vagus nerve stimulation (VNS) therapies from preclinical studies to human clinical applications (e.g., for stroke, heart failure, and inflammatory diseases) did not account for individual- or species-specific differences in nerve responses when selecting stimulation parameters. Lack of explicit consideration for producing equivalent nerve responses could contribute to clinical outcomes not replicating promising results from preclinical animal studies.

**Methods:**

We used models of VNS built with ASCENT (Musselman, PLoS Comput Biol 17:e1009285, 2021) to quantify nerve responses across species and simulate translation of VNS therapies via either recycling or linear scaling of stimulation parameters. For humans (*n* = 9) and pigs (*n* = 12), we used previously validated computational models with the standard clinical helical cuff electrode on individual-specific nerve morphologies (Musselman, J Neural Eng 20:acda64, 2023b). We also modeled rat VNS (*n* = 9) with the Micro-Leads Neuro bipolar cuff. We calculated thresholds for fiber activation (A-, B-, and C-fibers) with biphasic rectangular pulses (0.13, 0.25, 0.5 ms). We defined “K” as the ratio of activation thresholds between a pair of individuals. We used a mixed model ANOVA on the natural logarithm of K to test for differences in inter-species Ks across fiber types and pulse widths. Lastly, using the same nerve morphologies and application-specific device design (cuff and waveform), we developed models to predict nerve responses in chronic human and rat VNS studies for treatment of stroke, inflammation, and heart failure.

**Results:**

Depending on the individual and species, the activation amplitude required to produce a given nerve response varied widely. Thus, applying the same VNS parameters across individuals within a species produced a large range of nerve responses. Further, applying the same or linearly scaled stimulation amplitudes across species also produced highly variable responses. Ks were greater for B fibers than A fibers (*p* < 0.0001) and decreased with longer pulse widths (*p* < 0.0001 between consecutive pairs).

**Conclusions:**

The results highlight the need for systematic approaches to select stimulation parameters that account for individual- and species-specific differences in nerve responses to stimulation. Such parameter tuning may lead to higher response rates and greater therapeutic benefits from VNS therapies.

**Supplementary Information:**

The online version contains supplementary material available at 10.1186/s42234-025-00174-9.

## Background

Despite promising preclinical studies of vagus nerve stimulation (VNS) to treat a range of diseases, translating therapies to the clinic remains a challenge (Birmingham et al. 2014). Appropriate choice of VNS parameters to activate specific fibers and minimize off-target activation is required to achieve intended clinical outcomes and minimize side effects, respectively (Musselman et al. 2019). However, a systematic approach for selecting stimulation parameters that achieve targeted nerve responses is lacking, and promising VNS therapies often do not translate robustly to patients.

Species-specific differences in nerve morphology likely contribute to distinct patterns of nerve fiber activation from the same VNS parameters. Histology of the vagus nerve shows large differences in nerve morphology across species (Aristovich et al. 2021; Blanz et al. 2023; Fazan & Lachat 1997; Hammer et al. 2015, 2018; Licursi de Alcântara et al. 2008; Nicolai et al. 2020; Pelot et al. 2020d; Seki et al. 2014; Settell et al. 2020; Stakenborg et al. 2020; Verlinden et al. 2016). Human and pig vagus nerves are ~ 10× larger than rat vagus nerves, and the number and sizes of fascicles vary across species, e.g., 1 fascicle in rat, ~ 47 in pig, ~ 7 in human (Pelot et al. 2020d). However, VNS parameters applied in animal studies are often “translated” directly to clinical trials, e.g., VNS for stroke rehabilitation and heart failure, by applying the same stimulation intensities, and these parameters may not produce equivalent evoked responses.

In VNS for stroke rehabilitation, the stimulation parameters used in rat studies (Hays et al. 2016; Khodaparast et al. 2013; Porter et al. 2012; Pruitt et al. 2021) were recycled for use in patients (Dawson et al. 2021): biphasic rectangular waveform, 0.8 mA, 0.1 ms/phase. Following a motor cortical lesion, all rats that received VNS and rehabilitative training improved forelimb function (i.e., coordination and peak force in a lever grasp and pulling task) (Hays et al. 2016), but with identical stimulation parameters, only ~ 50% of patients that received VNS and rehabilitative training achieved clinically meaningful improvement (Dawson et al. 2021).

Similarly, despite promising data for VNS to treat heart failure in rats (Li et al. 2004) and canines (Sabbah et al. 2005, 2007; Vanoli et al. 1991; Zhang et al. 2009), pivotal clinical trials failed to achieve endpoints or were terminated prematurely (Anand et al. 2020; Gold et al. 2016; Konstam et al. 2019; Musselman et al. 2019). VNS consistently evoked bradycardia in the preclinical studies, which indicates activation of higher threshold B fibers (Yoo et al. 2013), but did not evoke bradycardia in the clinical studies (De Ferrari et al. 2017; Gold et al. 2016; Premchand et al. 2014; Sharma et al. 2021). Rather, in most patients the stimulation amplitude was limited by activation of Aα fibers, which produced intolerable laryngeal muscle activation, and heart rate changes from B fiber activation were not observed. Therefore, despite increasing the stimulation amplitude from preclinical experiments for use in humans, the amplitudes delivered clinically did not evoke responses comparable to those that treated heart failure in animals.

Computational models may inform proper selection of VNS parameters to achieve consistent nerve responses across individuals and thereby enable more successful translation of therapies. Previously, computational models of VNS enabled quantification of species- and individual-specific differences in patterns of nerve fiber activation due to nerve morphology (Aristovich et al. 2021; Blanz et al. 2023; Helmers et al. 2012; Musselman et al. 2023b). Validated computational models of VNS across humans, pigs, and rats showed differences in thresholds across species, and the models captured experimentally-measured inter-individual variability in nerve responses by modeling individual-specific nerve morphologies (Musselman et al. 2023b). Large differences in VNS thresholds were observed across species, consistent with large differences in nerve diameter, as well as number, size, and position of fascicles (Pelot et al. 2020d).

Achieving targeted nerve responses across species and individuals will likely improve success in translating VNS therapies to the clinic and in understanding mechanisms of action. Herein, using computational models, we show that applying stimulation parameters that achieved targeted nerve responses in one species failed to produce the same response in another species. Further, the same stimulation parameters applied across individuals of a given species also result in varied neural responses. We found a prohibitively large range of linear scaling factors to translate nerve responses across species (i.e., “translational scaling”) due to inter-individual variability in nerve morphology. Lastly, we developed models that mimic device and parameter settings used in preclinical and clinical studies of VNS for heart failure (Li et al. 2019; Premchand et al. 2014), inflammation (Kin et al. 2021; Koopman et al. 2016), and recovery from stroke (Dawson et al. 2021; Porter et al. 2012), and we used these models to predict the fiber types activated to produce the corresponding therapeutic responses.

## Methods

### Finite element models

We implemented finite element models (FEMs) of human, pig, and rat VNS using ASCENT (Musselman et al. 2021) with COMSOL Multiphysics v5.6.

**Fig. 1:**
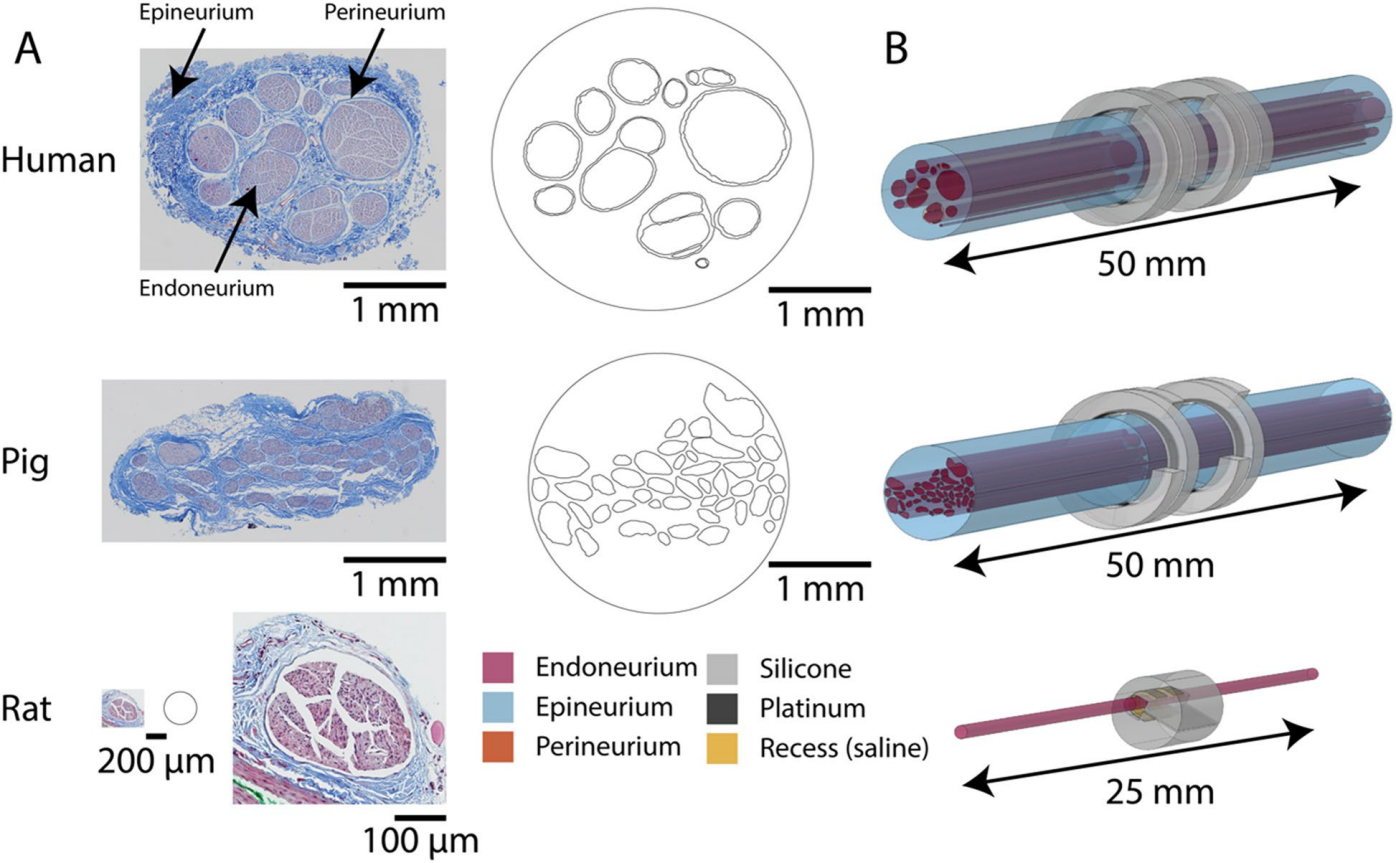
Histology-based computational models of vagus nerve stimulation (VNS) using individual-specific nerve morphology for humans, pigs, and rats. **A** Example histology and digitized nerve maps for human (Pelot et al., 2022a), pig (Pelot et al., 2022b), and rat (Pelot et al., 2022c) vagus nerves to show differences between species. Digitized nerve maps for all human and pig samples are available in (Musselman et al., 2023b), Fig. 1b and Supplemental Fig. 7f, respectively. We included an enlarged image of the rat nerve histology for clarity; the left-hand rat image is at the same scale as the human and pig histology. **B** Example finite element models of human, pig, and rat VNS using cuff electrodes that are used clinically (LivaNova helical cuff for pigs and humans) or used in preclinical studies (Micro-Leads Neuro for rats)

We first modeled acute VNS with the helical LivaNova cuff on the human and pig nerves and the Micro-Leads Neuro cuff on the rat nerves to investigate within-species neural responses and stimulation parameters for translation across species. We used published and validated models of human (*n* = 9) and pig (*n* = 12) VNS built with the ASCENT platform (v1.1.1) (Musselman et al. 2021, 2023a, 2023b) using segmented vagus nerve histology (Pelot et al. 2020a, 2020b, 2020d) and the clinical LivaNova bipolar helical cuff (LivaNova PLC, London, UK). We modeled rat VNS (*n* = 9) using ASCENT (v1.2.1) with cylindrical nerves matching the cross-sectional area of segmented rat vagus nerve histology (Pelot et al. 2020c, 2020d). We used the same methods as in a previously validated model of rat VNS with the 400 μm diameter Micro-Leads Neuro (Somerville, MA, USA) bipolar cuff (Musselman et al. 2023a, 2023b), but we modeled the perineurium as a surface impedance using the average thickness measured from each nerve’s histology: we calculated the difference in radii between equi-area circles for the outer and inner perineurium boundaries. We modeled each vagus nerve by extruding the 2D morphology (Fig. 1), and we centered the cuff along the length of the nerve (humans: 50 mm, pigs: 50 mm, rats: 25 mm).

Using the same nerve models as above, we simulated the cuff electrodes from published chronic human and rat VNS studies for treatment of stroke, inflammation, and heart failure (Fig. 7, Table 1). A helical cuff was implanted in human studies of VNS to treat stroke sequelae (Dawson et al. 2021) (MicroTransponder Inc., Lakeway, TX, USA), inflammation (Koopman et al. 2016) (LivaNova), and heart failure (Premchand et al. 2014) (LivaNova); therefore, we used the models of chronic VNS with the helical cuff on nine human nerves (Musselman et al. 2023a, 2023b). On the same human nerves, we also modeled cuffs used in investigational clinical studies of VNS. We modeled the ReStore system being used in a study of VNS to enhance recovery of motor and sensory function after neurological injury (Faltys et al. 2013) (NCT04534556) (Appendix 1 – ReStore). We also modeled the SetPoint Medical (Valencia, CA, USA) system used in a study of VNS to treat inflammation (Genovese et al. 2020) (Appendix 2 – SetPoint). Details for how the Restore and SetPoint Medical cuff diameters were chosen for the models are described in (Appendix 3 – Nerve Deformation for ReStore and SetPoint). Lastly, we modeled rat VNS for treatment of stroke (Porter et al. 2012), inflammation (Kin et al. 2021), and heart failure (Li et al. 2019) based on published custom bipolar cuff descriptions and dimensions (Appendix 4 – Finite Element Models of Rat VNS for Stroke, Inflammation, and Heart Failure) for the rat vagus nerve morphology in (Musselman et al. 2023b; Pelot et al. 2020d) (i.e., monofascicular nerve, circle with diameter of 235 μm).

**Table 1:** Overview of clinical and rat studies modeled to quantify nerve responses in VNS for stroke sequelae, inflammation, and heart failure

	**Stroke**	**Inflammation**	**Heart failure**
Human	(Dawson et al. 2021): chronically implanted helical cuff (MicroTransponder) ASCENT v1.1.1 (Fig. 7A) (Sivaji et al. 2019): chronically implanted ReStore cuff ASCENT v1.1.2 (Fig. 7D)	(Koopman et al. 2016): chronically implanted helical cuff (LivaNova) ASCENT v1.1.1 (Fig. 7B) (Genovese et al. 2020): chronically implanted SetPoint Medical cuff ASCENT v1.1.2 (Fig. 7E)	(Premchand et al. 2014): chronically implanted helical cuff (LivaNova) ASCENT v1.1.1 (Fig. 7C)
Rat	(Porter et al. 2012): chronically implanted custom bipolar cuff ASCENT v1.1.1 (Fig. 7G)	(Kin et al. 2021): chronically implanted custom bipolar cuff ASCENT v1.1.1 (Fig. 7H)	(Li et al. 2019): chronically implanted custom bipolar cuff ASCENT v1.1.1 (Fig. 7I)

We followed the methods of previously validated models for simulating acute and chronic VNS (Musselman et al. 2023a, 2023b); we used the acute models to analyze scaling of neural responses across species, and we used the chronic models to analyze specific preclinical and clinical studies with long-term cuff implants.

For acute models, we included a thin saline layer over all external surfaces of the cuff (100 μm for humans and pigs, 10 μm for rats) (Musselman et al. 2023a). We expanded the cuff diameter if needed to maintain the same saline layer thickness on the internal surface and accommodate the nerve diameter; for nerves with diameter smaller than the cuff diameter and twice the saline thickness, the remaining volume was modeled as saline. For the chronic models, we included the same saline layers on the internal and external surfaces, around which we added a layer of encapsulation tissue (250 μm for humans and pigs, 25 μm for rats) (Musselman et al. 2023a); for nerves smaller than cuff diameter minus twice the saline and encapsulation tissue thicknesses, the remaining volume was modeled as encapsulation tissue.

All conductivities and references are provided in Table 2. We assigned electrical conductivities to the endoneurium, epineurium, and skeletal muscle surrounding the nerve (Fig. 1). For fascicles with a single endoneurium bundle, we modeled the perineurium using a surface impedance (R_m_, Ω⋅m^2^) with thickness (thk_peri_, m) defined by the difference in radii of the effective area circles for the outer and inner perineurium boundaries:

**Table 2:** Material conductivities used in finite element models of VNS, adapted from Table 1 of (Musselman et al. 2023b)

Material	Electrical Conductivity σ (S/m)	References
Muscle	{0.086, 0.086, 0.35}	(Gielen et al. 1984)
Silicone	10^–12^	(Callister & Rethwisch 2012)
Platinum	9.43 × 10^6^	(de Podesta et al. 1996)
Saline	1.76	(Horch 2017)
Encapsulation Tissue	0.159	(Grill & Mortimer 1994)
Epineurium	0.159	(Grill & Mortimer 1994; Pelot et al. 2017; Stolinski 1995)
Perineurium	0.0008703	(Pelot et al. 2019; Weerasuriya et al. 1984)
Endoneurium	{0.167, 0.167, 0.571}	(Pelot et al. 2019; Ranck & BeMent 1965)

1 $${R}_{m}=\frac{th{k}_{peri}}{{\sigma }_{peri}}$$

For fascicles with multiple endoneurium bundles (i.e., fascicles with one or more intrafascicular perineurial septa), we meshed the perineurium as a conductive domain with finite thickness and conductivity (S⋅m^−1^). We assigned the electrical conductivity of silicone to the cuff substrate, platinum to the contacts, saline surrounding the cuff, and encapsulation tissue in models that represent chronic preclinical or clinical studies.

We modeled a point current source at the center of each platinum contact (Pelot et al. 2018). The outer boundaries of the models were grounded to represent an implanted pulse generator or subdermal needle serving as the counter electrode. We meshed the models with tetrahedral elements, and we solved the models using quadratic shape and solution shape functions. We solved Laplace’s equation for the electric potentials when each contact delivered 1 mA and the potential on the other contact was floating, which defined our solution bases; we then calculated the electric potentials for bipolar stimulation using superposition.

### Nerve fiber models

We simulated multi-compartment nerve fibers in NEURON v7.6 (Hines & Carnevale 1997). Histograms of the distributions of fiber diameters in the vagus nerve of multiple mammalian species have comparable modes and ranges (Agostinu et al. 1957; Asala & Bower 1986; Fazan & Lachat 1997; Guo et al. 1987; Licursi de Alcântara et al. 2008; Schnitzlein et al. 1958; Soltanpour & Santer 1996). Therefore, we used the same nerve fiber models to represent A fibers (13 μm diameter fibers) and B fibers (3 μm diameter fibers) across our models of human, pig, and rat nerves. We used the McIntyre-Richardson-Grill (MRG) model of mammalian myelinated fibers, as implemented in ASCENT, which enables simulation of arbitrary fiber diameters (McIntyre et al. 2002, 2004; Musselman et al. 2021). In our human models, we simulated additional MRG fiber diameters (4–12 μm) to show differences in concomitant fiber activation across individuals at a consistent A fiber activation level. Additionally, we modeled C fiber thresholds using the “parent” section of the Tigerholm model of unmyelinated mammalian fibers (Pelot et al. 2021; Tigerholm et al. 2014) with 0.8 μm diameter in human and rat nerves.

Our human models had 1–15 fascicles per nerve; we modeled one C fiber at the centroid of each fascicle, and we modeled myelinated fibers within each fascicle at random locations with a uniform density of 0.0001 fibers/μm^2^, resulting in 54–238 fibers per nerve and 1–139 fibers per fascicle. Our pig models had 33–63 fascicles per nerve; we modeled one C fiber at the centroid of each fascicle, and we modeled myelinated fibers within each fascicle at random locations with a uniform density of 0.0001 fibers/μm^2^, resulting in 130–247 fibers per nerve and 1–20 fibers per fascicle. Our rat models were monofascicular; we modeled 50 fibers randomly placed within each nerve for both myelinated and unmyelinated fibers. For a given nerve, we modeled myelinated fibers of different diameters at consistent locations in the nerve cross section. For a given fiber diameter, previous modeling studies showed that fiber location within a fascicle cross section has negligible effects on thresholds (Davis et al. 2023; Pelot et al. 2017). Therefore, single fibers positioned at the fascicle centroid were used. For the myelinated fibers in all models, we randomly offset the longitudinal fiber placement by half the internodal length in either direction to vary the alignment of the nodes of Ranvier with the electrode.

### Stimulation waveforms

We applied the extracellular potentials as a time-varying signal (“waveform”) to the fiber models in NEURON. In our analysis of translational scaling, we compared activation thresholds in response to single-pulse waveforms with three different biphasic symmetric rectangular pulse widths (0.13, 0.25, and 0.5 ms/phase). We calculated thresholds for additional pulse widths as needed to match the waveforms used in the clinical and preclinical studies of VNS (Table 3).

**Table 3:** Stimulation waveform and amplitudes delivered in the clinical and preclinical studies of VNS that we modeled

	**Stroke**	**Inflammation**	**Heart failure**
Human	(Dawson et al. 2021): Vivistim System IPG Biphasic pulses, first phase rectangular 0.1 ms/phase 0.8 mA (Sivaji et al. 2019): ReStore IPG Rectangular biphasic pulses 0.1 ms/phase 0.8 mA	(Koopman et al. 2016): Cyberonics IPG (now LivaNova) Biphasic pulses, first phase rectangular 0.5 ms/phase 1 mA (Genovese et al. 2020): SetPoint IPG Rectangular biphasic pulses 0.25 ms/phase 1.52 mA (mean)	(Premchand et al. 2014): LivaNova IPG Biphasic pulses, first phase rectangular 0.25 ms/phase 1.5–3 mA
Rat	(Porter et al. 2012): Pulse generator not reported Rectangular biphasic pulses 0.1 ms/phase 0.8 mA	(Kin et al. 2021): SAS-200 (Unique Medical Co., Ltd., Tokyo, Japan) Rectangular biphasic pulses 0.5 ms/phase 0.1, 0.25, 0.5, 1 mA	(Li et al. 2019): ISE1000SA, Unimec Inc., Tokyo, Japan) Rectangular biphasic pulses 0.2 ms/phase 0.1, 0.13 mA

For our human VNS models, we approximated the secondary phase of the waveform to be rectangular; the current amplitude and time constant (if passive) of the recharge phase of the waveform produced by the Vivistim, Cyberonics, and LivaNova devices have not been disclosed (Dawson et al. 2021; Koopman et al. 2016; Premchand et al. 2014). In the same human VNS models, (Musselman et al. 2023b) reported that thresholds were only ~ 20% lower when a monophasic rather than biphasic rectangular waveform was used.

### Calculation of fiber thresholds

We calculated activation thresholds for each fiber using a bisection search algorithm with a tolerance of 1%. To prevent detecting an ohmic rise in transmembrane voltage rather than propagating action potentials, we detected action potentials at 90% of the fiber length. We saved the action potential time at 10 and 90% fiber length and confirmed bidirectionally propagating action potentials in all simulations at threshold amplitude.

### Quantification of nerve responses

We quantified the relationship between stimulation amplitude and activation level for each fiber type. We determined thresholds to activate 20, 50, 80, and 100% of A and B fibers in each nerve (I_20_, I_50_, I_80_, and I_100_). In human and pig models, which we randomly populated with a uniform density of fibers across fascicles, and in rat models, which we randomly populated with a set number of fibers, we used the percentile of each fiber threshold to determine the amplitude corresponding to each activation level; thus, each fiber threshold contributed equally to the calculation of nerve response.

### Scaling factors to translate nerve responses

We computed the K ratio of thresholds for each activation level and fiber type (i.e., 20, 50, 80, or 100% of A or B fibers are active in a given nerve) for all pairs of individuals across and within species. For example, the ratio of currents to activate 20% of A fibers in pig “n” versus rat “m” was calculated as:

2 $$K_{20A,\;Rat\#m\rightarrow Pig\#n}=\frac{I_{20A,\;Pig\#n}}{I_{20A,\;Rat\#m}}$$

We ran a mixed model ANOVA in JMP® Pro 17.2 (SAS Institute, Cary, NC, USA) on the natural logarithm of K ratios between individuals from smaller to larger species (rat to pig: 108 Ks (9*12), rat to human: 81 Ks (9*9), pig to human: 108 Ks (12*9)). We used a natural logarithm transformation because the data have a lower bound of 0, and the scaling factors spanned orders of magnitude. We included fixed effects of species pair, fiber type (A and B fibers), pulse width (0.13, 0.25, and 0.5 ms/phase), and activation level (i.e., I_20_, I_50_, I_80_, and I_100_), and all interaction terms. We included a random effect of paired individuals across species.

## Results

### Differences in activation thresholds within and across species

**Fig. 2:**
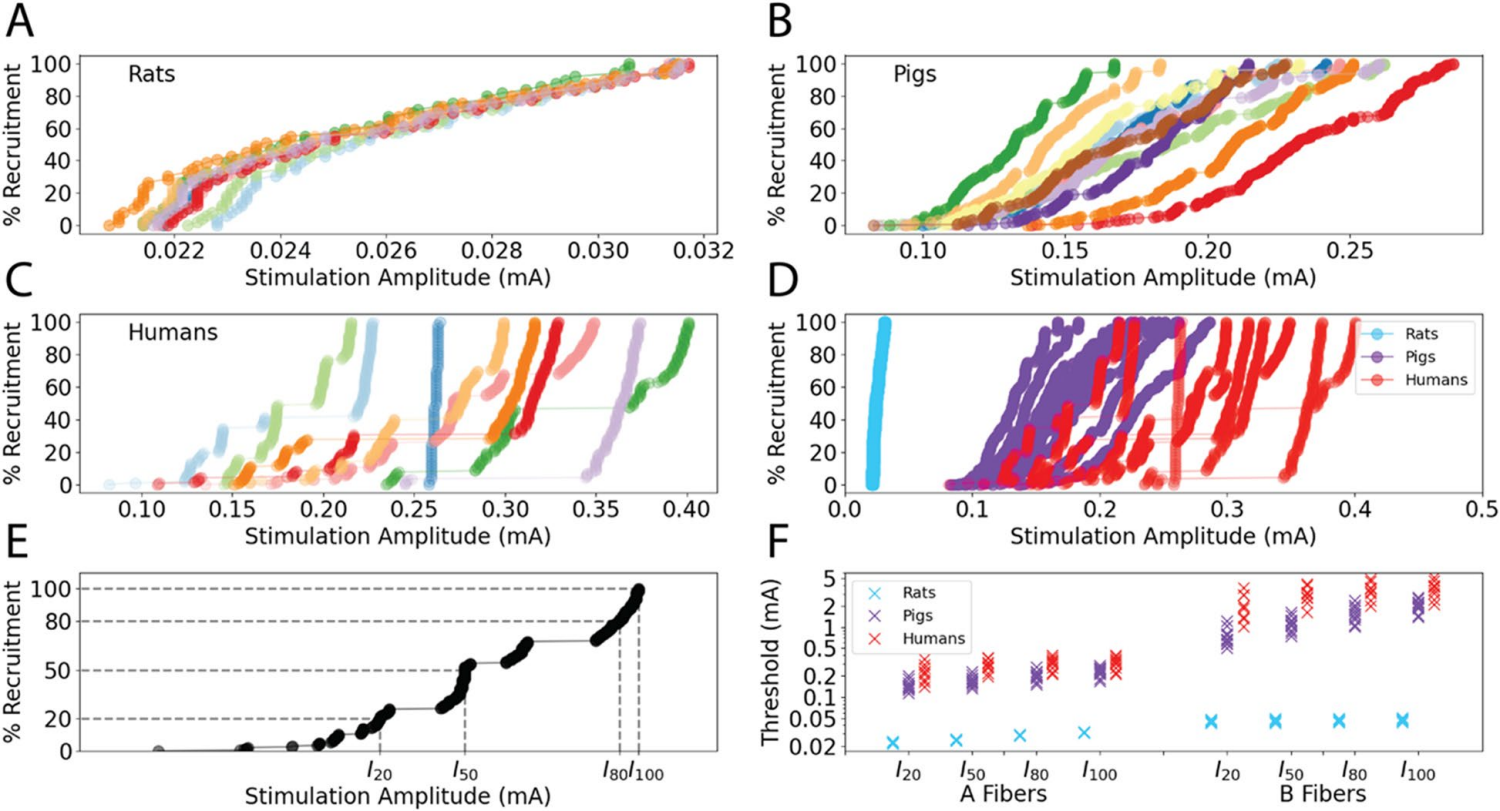
Stimulation amplitudes to activate fibers from individual-specific models of acute VNS in populations of rat, pig, and human nerves with 0.25 ms/phase biphasic rectangular waveform. **A** Dose–response curves for A fibers in rat nerves (*n* = 9). **B** Dose–response curves for A fibers in pig nerves (*n* = 12). **C** Dose–response curves for A fibers in human nerves (*n* = 9). Data for each nerve have a different color in panels (**A**-**C**). **D** Dose–response curves from panels (**A**-**C**) for A fibers with 0.25 ms/phase biphasic rectangular waveform. **E** Example dose–response curve for A fibers in a human model to show calculated threshold currents for each activation level (i.e., I_20_, I_50_, I_80_, and I_100_). **F** I_20_, I_50_, I_80_, and I_100_ threshold currents for A and B fibers in populations of humans, pigs, and rats. Each marker is a different individual

Stimulation amplitudes that activated fixed proportions of nerve fiber types varied across individuals of a given species (Fig. 2A-C). Activation amplitudes for humans were slightly higher than for pigs, but the amplitude ranges overlapped for each fiber type (Fig. 2D, F). Notably, human and pig threshold amplitudes were ~ 10–100 × higher than rat amplitudes depending on fiber diameter and activation level (Fig. 2D, F). Additionally, as seen in the discontinuous dose–response curves for humans (i.e., large jumps in current required to increase recruitment) (Fig. 2C), we observed more pronounced fascicular recruitment for humans than rats or pigs (Fig. 2A-B).

**Fig. 3:**
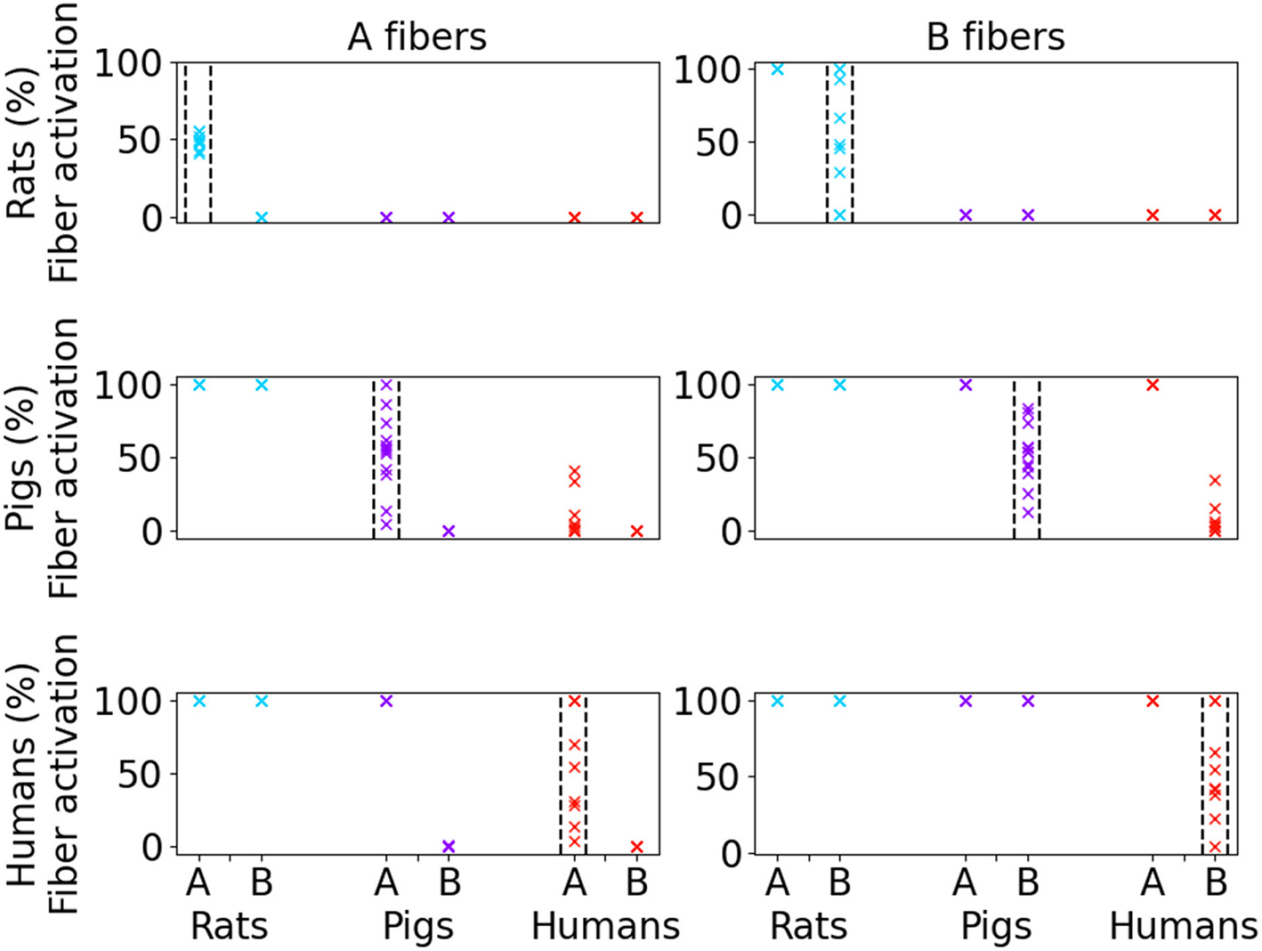
Percent of A fibers and B fibers activated in rats, pigs, and humans (labels along the bottom) in response to stimulation with the mean I_50_ from each species (rows, labels on left) and fiber type (columns, labels at the top). Each data point represents the percent of A or B fibers activated for an individual. All data shown are for 0.25 ms pulse width for acutely implanted cuff. The vertical dashed lines delineate the responses that match the species and fiber type of the delivered mean I_50_

There were no overlaps in the amplitude ranges of A and B fiber thresholds with 0.25 ms pulse width (Figs. 2F, 3), except for two pigs which each had a very small fascicle at the nerve boundary yielding some B fiber activation at amplitudes that were lower than the highest A fiber activation amplitudes. The range of amplitudes to activate 50% of fibers (I_50_) with a symmetric biphasic rectangular waveform (0.25 ms/phase) was larger for humans (A fibers: coefficient of variation (CV) = 0.19; B fibers: CV = 0.2) and pigs (A fibers: CV = 0.15; B fibers: CV = 0.22) than for rats (A fibers: CV = 0.02; B fibers: CV = 0.04).

More generally, looking at fiber diameters from 3 to 13 μm in 1 μm increments (Appendix 5 – Concomitant Fiber Responses with Activation of 50% of A Fibers), the stimulation amplitude that produced a targeted nerve response (e.g., 50% of 13 µm A fibers in human nerves) resulted in a range of concomitant activation of other fiber diameters depending on the individual, due to differences in nerve morphology (e.g., fibers as small as 7 μm in human #540 versus fibers as small as 11 μm in human #520).

### Adjustment of stimulation parameters is required to achieve equivalent nerve responses

The range of activation thresholds across individuals and across species (Fig. 2) suggested that applying the mean I_50_ for A or B fibers across all individuals of a species will not produce equivalent responses. Indeed, the mean I_50_ for a given species and fiber type did not typically activate 50% of fibers, and we observed a large range of individual responses: 41–56% A and 0–100% B fibers in rats, 4–100% A and 13–84% B fibers in pigs, and 4–100% A and 4–100% B fibers in humans (Fig. 3, data within dashed lines). Therefore, to achieve equivalent nerve responses within a species, stimulation amplitude must be adjusted to accommodate individual differences in nerve morphology.

Applying the mean I_50_ from one species did not produce an equivalent nerve response in another species. Delivering the mean I_50_ for A or B fibers from rats produced no activation of A or B fibers in either pigs or humans (Fig. 3, top row, purple and red). Conversely, the mean I_50_ for A or B fibers from pigs or humans activated all A and B fibers in rats (Fig. 3, bottom two rows, blue). The mean I_50_ for A or B fibers in pigs consistently resulted in less than 50% activation of the same fiber type in humans (Fig. 3, middle row, red), and the mean I_50_ for A or B fibers in humans consistently resulted in 100% activation of the same fiber type in pigs (Fig. 3, bottom row, purple). These findings are consistent with the data in Fig. 2D, where rat thresholds were much lower than pig and human thresholds, and pig thresholds trended lower or comparable to human thresholds.

### Range of scaling factors across species is prohibitively large

**Fig. 4:**
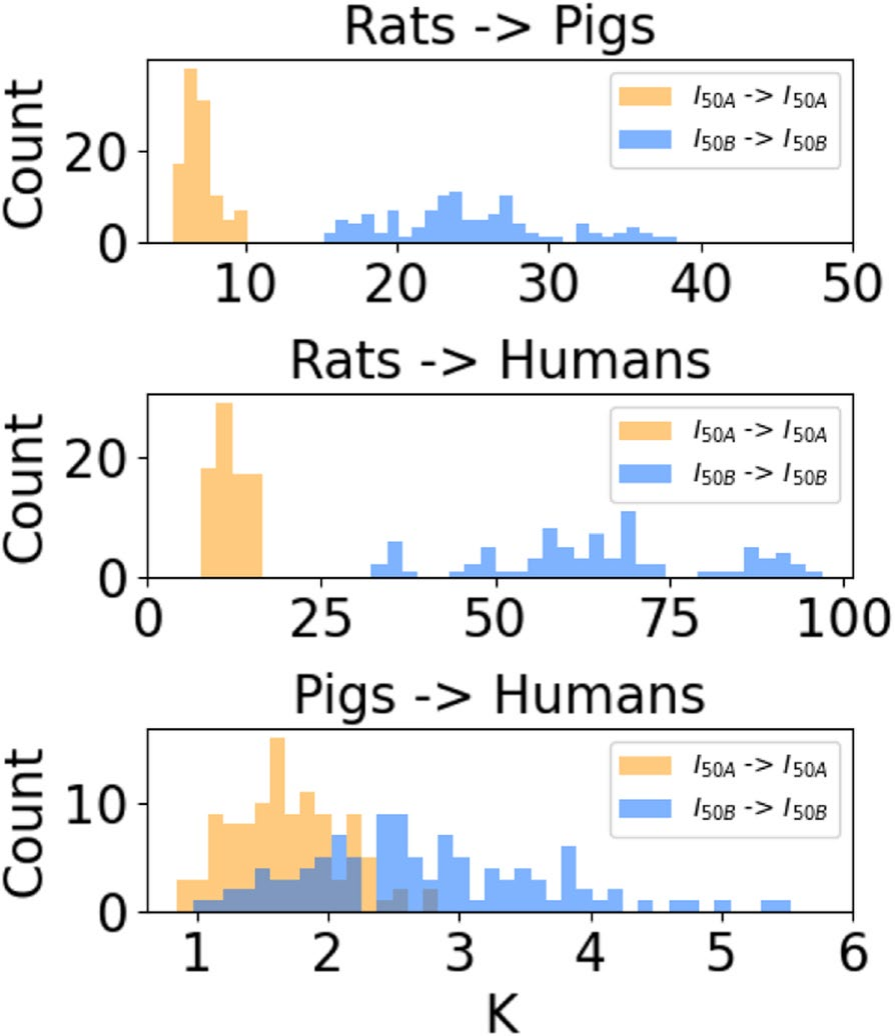
Histograms of linear scaling factors (K) between stimulation amplitudes that activate 50% of nerve fibers (i.e., I_50_ for A or B fibers) between all pairs of all individuals of different species with 0.25 ms pulse width for acutely implanted cuff; data for reverse translation of 0.25 ms and additional pulse widths (0.13 ms and 0.5 ms) are provided in Appendix 6 – Histograms of Linear Scaling Factors for Additional Pulse Widths

We assessed the performance of linear scaling of stimulation parameters between species to account for species-specific differences in nerve responses. We calculated the ratio between current amplitudes to activate 20, 50, 80, 100% of A or B fibers for all possible pairs of individuals between species (K ratio in Eq. 2). When scaling from smaller to larger species, Ks to scale I_50_ current amplitudes for A or B fibers spanned large ranges and the Ks were larger for B fibers than for A fibers (Fig. 4; Appendix 6 – Histograms of Linear Scaling Factors for Additional Pulse Widths): rats to pigs (A: 5.2–9.8, B: 15.3–38.4), rats to humans (A: 7.8–15.7, B: 33.2–96.9), and pigs to humans (A: 0.9–2.8, B: 1.0–5.5). Thus, consistent with Fig. 2F, larger Ks were required to scale current amplitudes from rats to humans than from pigs to humans.

A global ANOVA on the natural logarithm of the Ks from smaller to larger species revealed a significant interaction between species pair, fiber type, and pulse width, and therefore, we subdivided by species pair for ANOVAs on fiber type, pulse width, and their interaction term. Post-hoc Student’s t-tests showed that Ks were greater for B fibers than A fibers (*p* < 0.0001) and that Ks decreased with longer pulses width (*p* < 0.0001 between consecutive pairs).

**Fig. 5:**
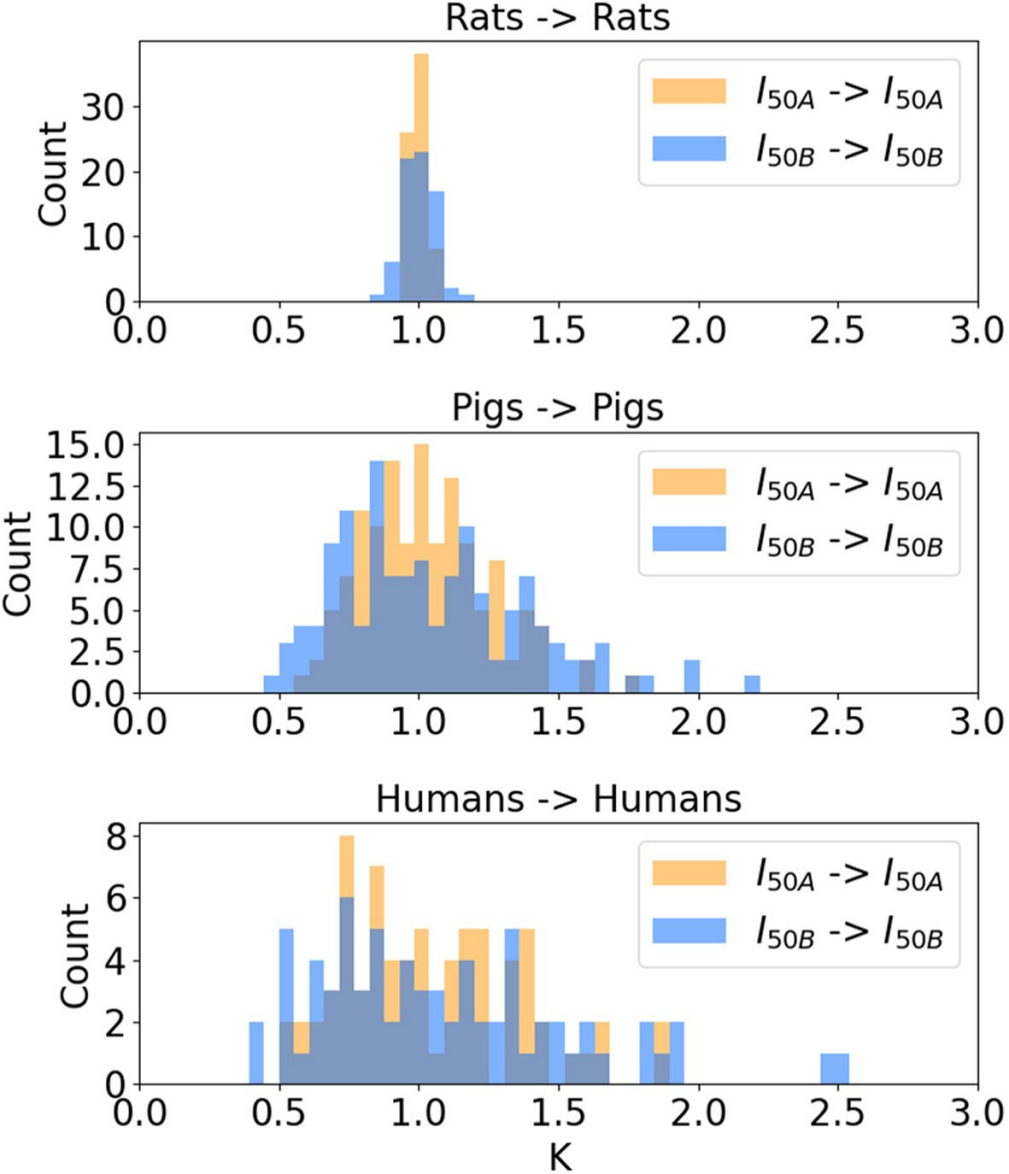
Histograms of linear scaling factors (K) between stimulation amplitudes that activate 50% of nerve fibers (i.e., I_50_ for A or B fibers) between all individuals of the same species in both directions for 0.25 ms pulse width for acutely implanted cuff. Each row is a different species

We quantified the variability of nerve responses within a species by plotting the Ks for 50% activation of A and B fibers between pairs of individuals from the same species (Fig. 5). There was substantial variability in Ks within each species, especially humans and pigs, which is expected given their more complex and variable nerve morphology. Since it is unknown where an individual activation amplitude falls within the distribution for an entire species, higher variability of within-species Ks will contribute to increased variance of Ks between species. Lastly, the Ks were comparable for small and large fibers, which contrasts with the between-species effects of fiber diameter (Fig. 4).

### Responses to linear scaling of amplitudes between species

**Fig. 6:**
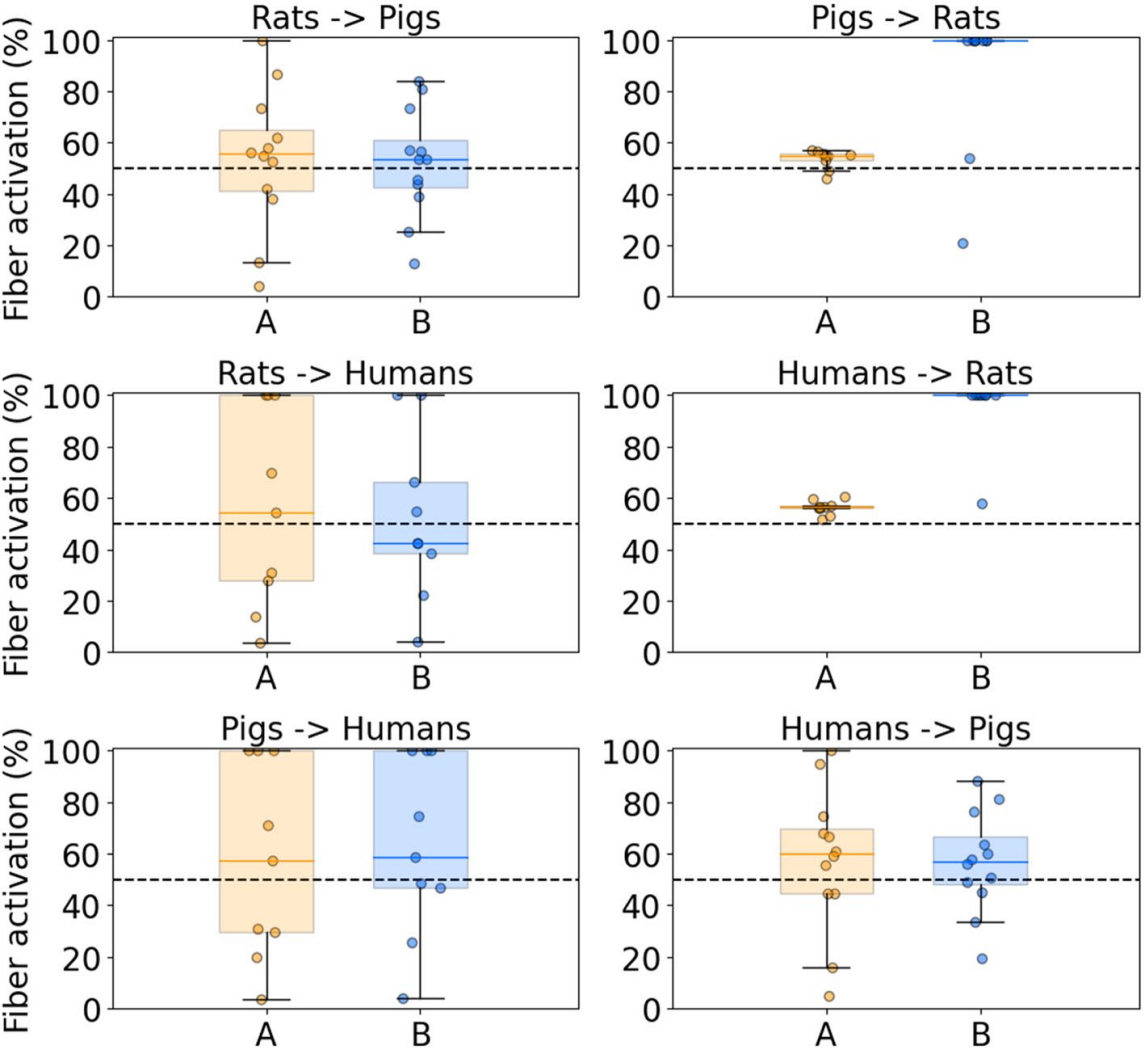
Responses to stimulation amplitudes determined by multiplying the mean I_50_ for A or B fibers in a given species by the mean linear scaling factor (K) of a given species pair, using a 0.25 ms/phase biphasic waveform for acutely implanted cuff. The left and right columns provide the Ks from smaller to larger species and from larger to smaller species, respectively. Each data point denotes the response of A (orange) or B (blue) fibers across each individual. The horizontal line denotes the median, the box denotes the upper (Q3) and lower (Q1) quartiles, and the upper and lower whiskers show Q3 + 1.5*(Q3-Q1) and Q1—1.5*(Q3-Q1), respectively

We simulated the responses to linear scaling of activation amplitudes between species, despite the large variability in Ks (Fig. 4). For A or B fibers, we multiplied the mean I_50_ of the starting species by the mean K for the species pair (i.e., “mean linear scaling”); we then applied the resulting scaled currents to the individuals in the ending species (Fig. 6).

Mean linear scaling of I_50_ produced the targeted nerve response on average, but there were exceedingly large ranges of responses across individuals. From rats to pigs, scaling currents for 50% activation produced ranges from 4–100% activation for A fibers and 13–84% for B fibers. Similarly, mean linear scaling from rats to humans produced ranges of 4–100% and 4–100% for A and B fibers. Lastly, mean linear scaling for 50% activation from pigs to humans produced 4–100% and 4–100% activation of A and B fibers. Scaling currents from humans or pigs to rats produced a very narrow range of responses, which reflects the monolithic rat nerve morphology and thus small inter-individual differences in activation amplitudes (Fig. 2A). Therefore, even though the target response was achieved on average, inter-individual differences within species prevented scaling from producing equivalent nerve responses across individuals when using the mean K to scale current amplitudes.

### Model-predicted nerve responses to VNS for new indications

We modeled published preclinical and clinical studies of VNS for stroke recovery, inflammation, and heart failure, using their cuff designs and stimulation pulse widths. We simulated thresholds of A, B, and C fibers in response to biphasic rectangular stimulation waveforms, and in all simulations, we observed bidirectionally propagating action potentials.

Our model thresholds for rats and humans were consistent with available in vivo and clinical data of VNS for stroke recovery, respectively, and suggest that stimulation amplitude was not sufficiently increased from rats to produce equivalent nerve responses in humans. VNS parameters for stroke recovery that were effective in rats (i.e., 0.8 mA, 0.1 ms/phase biphasic pulses) (Khodaparast et al. 2013; Porter et al. 2012; Pruitt et al. 2021) were applied without modification (“recycled”) in humans (Baylor Research Institute 2023; Dawson et al. 2021).

**Fig. 7:**
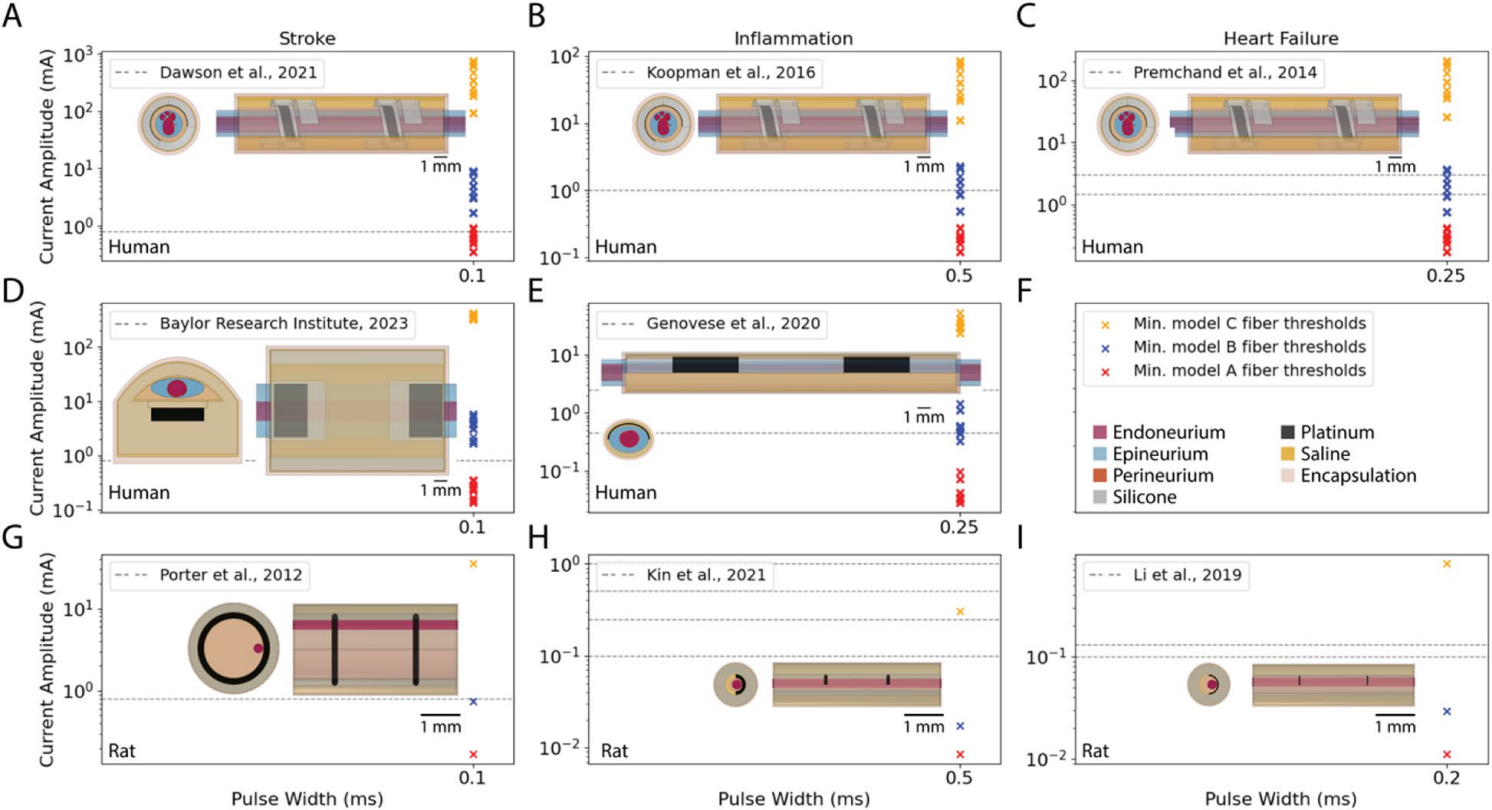
Comparison of model threshold currents for A (red), B (blue), and C (yellow) fibers with the rectangular biphasic pulse currents delivered in preclinical and clinical studies of VNS. Each panel shows the cuff geometry modeled to match the experimental setup and the lowest threshold in each nerve for each fiber type (colored x’s; legend in panel F). The experimental stimulation amplitudes are included in each panel (gray dashed lines). **A** (Dawson et al. 2021) delivered 0.8 mA in all patients. **B** (Koopman et al. 2016) delivered 1 mA in all patients in the TNF production study. **C** (Premchand et al. 2014) delivered 1.5–3 mA in patients (as marked) with a 2.0 ± 0.6 mA (mean ± SD). **D** (Baylor Research Institute 2023) delivered 0.8 mA in all patients. **E** (Genovese et al. 2020) delivered 0.45–2.5 mA in patients (as marked) with a 1.52 ± 0.55 mA (mean ± SD). **G** (Porter et al. 2012) delivered 0.8 mA in all animals. **H** (Kin et al. 2021) delivered different stimulation amplitudes (0.1, 0.25, 0.5, 1 mA) to each cohort of animals; 0.25 and 0.5 mA produced an anti-inflammatory effect, but 0.1 and 1 mA did not. **I** (Li et al. 2019) delivered 0.1–0.13 mA (as marked) in all animals

Recycled stroke parameters activated A fibers in rat models (Fig. 7G), human models (Fig. 7A, D), and in patients. In 2/53 patients, (Dawson et al. 2021) stimulation amplitude was reduced (to 0.7 and 0.6 mA) because 0.8 mA was intolerable, indicating the parameters produced activation of laryngeal muscles, a known side-effect from activation of A fibers in the cervical vagus nerve (Yoo et al. 2013). (Porter et al. 2012) did not demonstrate activation of A fibers in rats by either recording (laryngeal EMG) or reporting side-effects (retching) related to activation of laryngeal muscles. Further, the recycled stroke parameters activated B fibers in the rat models (Fig. 7G) but not in the human models (Fig. 7A, D). Neither (Porter et al. 2012) nor (Dawson et al. 2021) recorded signals to demonstrate activation of B fibers (e.g., heart rate changes with VNS (Yoo et al. 2013)). Lastly, no C fibers were activated in rat or human models with these parameters, and data are not available to confirm if C fiber activation occurred in the in vivo or clinical study.

VNS parameters used clinically for inflammation (Koopman et al. 2016) with the LivaNova cuff matched the highest amplitude delivered in rats (Kin et al. 2021) with the same pulse width (i.e., 1 mA, 0.5 ms/phase biphasic pulses) (Fig. 7B, H). These parameters activated A fibers in both rat and human models, B fibers in rat and in 3/9 human models, and C fibers in rat models with the highest two amplitudes (0.5 and 1 mA) but did not activate C fibers in human models (Fig. 7B, H). In the SetPoint clinical study of VNS for inflammation (Genovese et al. 2020), a shorter 0.25 ms pulse width was delivered and activated A and B fibers in all human models at mean amplitude (mean amplitude 1.52 mA; range 0–2.5 mA). (Kin et al. 2021) and (Genovese et al. 2020) did not mention activation of laryngeal muscles in rats or humans, respectively. (Koopman et al. 2016) reported a comparable, but not further described, level of side effects related to laryngeal muscle (A fiber) activation as previously published VNS studies for epilepsy in humans. None of (Kin et al. 2021), (Koopman et al. 2016) or (Genovese et al. 2020) reported heart rate changes (B fiber activation) with VNS.

VNS parameters used clinically for heart failure (Premchand et al. 2014) activated A fibers in all human models and B fibers in 3/9 and 6/9 human models for the lower (i.e., 1.5 mA) and upper-bounds (i.e., 3 mA) of current amplitude, respectively (Fig. 7C). In the clinic, the current amplitude was 2.0 ± 0.6 mA (59 patients, mean ± SD) (Premchand et al. 2014), but further increasing stimulation amplitude was limited by side effects related to laryngeal muscle (A fiber) activation in 50/59 patients and acute heart rate changes (B fiber activation) in 4/49, which is consistent with our model predictions of A and B fiber activation at this intensity. From baseline to six months, Holter monitor analysis showed modest reduction in heart rate (i.e., 3.9 ± 9.8 beats/min; 59 patients, mean ± SD), which is consistent with activation of B fibers in some models. The stimulation parameters used in rat studies (Li et al. 2019) activated both A and B fibers in our rat model but were below C fiber thresholds (Fig. 7I). (Li et al. 2019) did not record or report laryngeal muscle activation in rats, but stimulation usually produced bradycardia, consistent with B fiber activation in the rat model.

## Discussion

The selection of stimulation parameters to translate VNS therapies from preclinical animal studies to the clinic is challenging due to within- and across-species differences in nerve responses. Using quantitatively validated models based on individual-specific nerve morphologies of humans, pigs, and rats, we demonstrated that substantial differences in nerve fiber activation resulted when simply taking the amplitude used in one species and applying it to another (recycling). Further, linear scaling of amplitudes between species produced a broad range of nerve responses due to individual differences in nerve morphology. Our results highlight the need for species- and individual-specific selection of stimulation parameters, and morphologically realistic validated computational models provide a tool to determine the stimulation parameters required to evoke targeted nerve responses.

### Within and across species differences in nerve morphology affect thresholds

There is a complex relationship between nerve morphology, applied stimulation, and neuronal responses, and this relationship is captured by our models of VNS. Differences in morphology between individuals and species affected thresholds due to differences in electrode-fiber distance, fascicle diameter, and perineurium thickness. Human and pig vagus nerves have comparable diameters, but humans generally have fewer and larger fascicles that are more variable in diameter. Conversely, rat vagus nerves are monofascicular and ~ 10× smaller than human. The variability of the currents required for 50% activation of fibers across individuals was greatest in humans followed by pigs and rats, which parallels the differences in nerve morphology across individuals (Pelot et al. 2020d). Our models show that the uniform morphology of the rat vagus nerve results in low inter-individual variability in nerve activation (Fig. 2). As a result, preclinical studies in rats may not reflect the effects of inter-individual anatomical differences, as present in human VNS, that may be critical for successful clinical translation.

The Ks to scale current amplitudes between inter-species pairs of individuals were lower for A fibers than for B fibers when scaling from smaller to larger species, but the within-species Ks were similar for A and B fibers (Figs. 4, 5). The distribution of Ks to scale activation amplitudes for A and B fibers overlapped only slightly between pigs and humans, which both used the same cuff and had comparable electrode-fiber distances. These data indicate that, for example, stimulation amplitudes that activate the same proportion of A fibers in different species will activate differing proportions of other fiber types (e.g., B fibers).

### Implications for selection of stimulation parameters

Our models of VNS demonstrated that applying the same stimulation parameters across species did not achieve equivalent nerve responses, and differences in nerve fiber activation could contribute to the failed translation of VNS therapies to the clinic. Recycling stimulation parameters across species does not account for known nerve morphological differences, and this approach produces substantial differences in nerve responses between species.

Stimulation parameters that were determined to be effective in preclinical studies of VNS for stroke in rats (Khodaparast et al. 2013; Porter et al. 2012; Pruitt et al. 2021) were applied without modification in humans (Dawson et al. 2021) (i.e., 0.1 ms/phase; 0.8 mA). Although (Pruitt et al. 2021) demonstrated that the therapeutic benefits of VNS for stroke recovery in rats were sensitive to stimulation amplitude, no modifications were made in stimulation parameters to account for the pronounced differences between rat and human nerves. Average currents that activated 50% of A or B fibers in rats resulted in no activation in human nerves (Fig. 3). Therefore, while all rats with VNS demonstrated complete recovery to pre-lesion performance (Khodaparast et al. 2013), it is perhaps not surprising that only about half of stroke patients with VNS achieved a clinically meaningful response in Fugl-Meyer Assessment Upper Extremity scores (FMA-UE). Our application-specific models predicted that both A and B fibers were activated in rat VNS for stroke, but that the same stimulation parameters (i.e., 0.1 ms/phase; 0.8 mA) only activated A fibers in a subset of models of human VNS. These data indicate that the same VNS parameters applied across species and individuals produce variable patterns of nerve fiber activation, thereby making it challenging to achieve efficacy or to conclude which fibers are therapeutically necessary or sufficient.

Ongoing efforts to apply VNS for inflammatory diseases are translating stimulation parameters from rats to humans, albeit less explicitly. Clinical VNS studies to treat inflammatory diseases (Koopman et al. 2016) used stimulation parameters (0.5 ms/phase; 1 mA) equal to or higher than those used in rats (0.5 ms/phase; 0.1, 0.25, 0.5, or 1 mA) (Kin et al. 2021) (Fig. 7B, H). Our models indicated that the parameters used in rat experiments for inflammation activated A fibers, B fibers, and, at the highest amplitudes (i.e., 0.5 and 1 mA), C fibers, but that clinical stimulation parameters activated A and B fibers in 9/9 and 3/9 individuals, respectively, and C fibers were not activated in any individuals. (Kin et al. 2021) demonstrated that anti-inflammatory effects in rats were only present at the intermediate amplitudes used (i.e., 0.25 and 0.5 mA); in our models, 0.25 mA activated A and B but not C fibers, and 0.5 mA activated A, B, and C fibers. At the lowest amplitude used (0.1 mA), our models indicated that A and B fibers were still activated. Therefore, our models suggest that VNS parameters that produce activation of fibers with excitability close to C fibers may be necessary to produce the full effect of VNS to treat inflammatory diseases.

### Individual-specific differences preclude translation of nerve responses with linear scaling factors

Our models produced a wide range of responses across individuals when using linear scaling of VNS parameters across species, and these differences in nerve fiber activation may contribute to the failed translation from preclinical to clinical studies of VNS for heart failure. Linear scaling of stimulation parameters across species, even when the average scaling factor was known, did not produce equivalent nerve responses (Fig. 6). Preclinical studies indicated that stimulation amplitudes sufficient to produce bradycardia via activation of B fibers were necessary to produce therapeutic effects in heart failure (Musselman et al. 2019; Yoo et al. 2013). However, despite delivering much higher amplitudes (i.e., ~ 20–40 × larger) in human studies (Gold et al. 2016; Hauptman et al. 2012; Premchand et al. 2014) than in rats (Li et al. 2004), VNS did not consistently induce bradycardia in humans. Our models suggested that stimulation amplitudes ~ 30–100 × higher than in rats are required to produce B fiber activation in humans (Fig. 4). Indeed, the models indicated that preclinical amplitudes for heart failure in rats activated A and B fibers, while clinical amplitudes activated A fibers in all individuals but B fibers in only some individuals. Except for B fibers located in small superficial fascicles in our pig models, there were no overlaps between the threshold ranges of A and B fibers with 0.25 ms pulse width; therefore, with such circumneural cuff electrodes, assuming uniform distribution of fiber types in the nerve cross section, therapeutic B fiber activation cannot be achieved without maximal activation of side effect-causing A fibers.

### Opportunities to improve translation of VNS with targeted nerve responses

Selection of stimulation parameters to achieve equivalent nerve responses between individuals and across species is important to the continued advance of VNS therapies. Current practice for selecting VNS parameters depends on the intended therapeutic outcome, but in VNS to treat epilepsy, amplitude is increased as tolerated (i.e., limited by activation of A fibers innervating the larynx) until therapeutic effects are satisfactory. Depending on individual-specific nerve morphology and the spatial distribution of fibers associated with side effects versus therapeutic effects, this approach to patient programming is likely to produce differing patterns of nerve fiber activation across individuals. Higher response rates and greater therapeutic benefits may be obtained with optimized dosing of VNS in humans.

Selection of stimulation parameters could be improved by recording stimulation-evoked neural signals (e.g., compound action potentials (CAPs)) to determine the specific fiber populations activated, but this requires that the target fiber population’s conduction velocity be known. Critical for determining which fiber populations to target for therapy, animal experiments enable more flexibility than clinical studies to test systematically stimulation parameters and make detailed measurements of neural and physiological responses to stimulation, including therapeutic responses and side effects (Ahmed et al. 2020; Blanz et al. 2023; Chang et al. 2020; Huffman et al. 2019, 2023; Levine et al. 2014; Meregnani et al. 2011; Nicolai et al. 2020; Pelot & Grill 2020; Sabbah et al. 2007, 2005; Zhang et al. 2009).

Accurate nerve morphologies are required for individual-specific model-based selection of stimulation parameters, and advances in nerve imaging and mapping technologies are needed to develop patient-specific models. For example, in situ nerve imaging with knowledge of embedded fiber tractography to end-organs would be useful for selecting contact polarities or stimulation waveforms to activate selectively target fiber populations while minimizing off-target activation. Previous work demonstrated technical readiness to collect 3D nerve structure data from *excised* nerves using microCT (Thompson et al. 2020; Upadhye et al. 2022), and fiber tractography can be determined using dextran fluorescent tracers (Ravagli et al. 2020) or viral tracers (Wei et al. 2014). However, further advances in imaging will be needed to determine 3D nerve geometry and tractography in live animals and humans to determine nerve morphology. Alternatively, model-based design of electrodes and stimulation paradigms could overcome these knowledge gaps by leveraging closed-loop systems that respond to manual inputs from the patient or clinician, or to measured neural and physiological signals.

Targeting specific nerve responses to stimulation is complicated by inter-individual differences in nerve morphology, and these differences present challenges for translating VNS therapies to the clinic. Currently, fiber responses other than those producing intolerable side effects are not monitored or quantified in patients. Our results demonstrate that without predicting (e.g., models based on patient-specific imaging) or measuring (e.g., recording and characterizing evoked CAPs or biomarkers) individual responses to VNS, clinical devices are unlikely to produce a targeted nerve response. Therefore, VNS may require advanced tools to quantify and tune nerve responses to stimulation and guarantee that nerve responses meet patterns required for therapy.

Many prior efforts examined approaches to achieve more targeted stimulation of peripheral nerve fibers via design of electrode geometry and/or stimulation parameters, as reviewed in Table 1 of (Fitchett et al. 2021). Such design approaches could incorporate individual-specific differences in nerve morphology and functional organization of fibers (Jayaprakash et al. 2023; Kronsteiner et al. 2022; Settell et al. 2020). For example, (Aristovich et al. 2021; Blanz et al. 2023) used multicontact cuff electrodes to achieve spatial selectivity, demonstrated by targeting functionally distinct groups of fascicles in sheep and pig vagus nerves. As a complement to spatial selectivity, waveform design can enable selective fiber-type activation. For example, if conventional low-frequency stimulation is used to activate all fibers in the nerve, quasi-trapezoidal pulses or kilohertz-frequency signals can then be used for selective block of large myelinated fibers (Pelot & Grill 2020; Tosato et al. 2007).

Lastly, in addition to stimulation parameters, device design choice must be factored into therapies and translating stimulation-evoked nerve responses. Though not the focus of this work, our model thresholds show that a loosely fitting cuff electrode as in (Porter et al. 2012) will produce much higher thresholds on the same nerve than smaller diameter cuffs (Kin et al. 2021; Li et al. 2019) (Fig. 7G versus Fig. 7H-I).

### Limitations

Our models may underestimate the true range of activation amplitudes for specific nerve responses, and therefore our modeled responses to applying a mean current amplitude across individuals of a species (Fig. 3) and linear scaling of parameters (Figs. 4, 6) are likely conservative estimates for the ranges of nerve responses in vivo. First, we modeled VNS for individual-specific nerve morphologies from nine humans, twelve pigs, and nine rats, and these samples may not be representative of the entire species’ populations. However, the nerves that we modeled (Pelot et al. 2020d) are mostly consistent in diameter and number of fascicles with other published nerve morphologies for humans (Hammer et al. 2015, 2018; Seki et al. 2014; Stakenborg et al. 2020; Verlinden et al. 2016), pigs (Stakenborg et al. 2020), and rats (Fazan & Lachat 1997; Licursi de Alcântara et al. 2008). The accuracy of thresholds predicted by the models could be improved with information about the fluid thickness between the cuff and the nerve, particularly for human and pig nerves; thresholds increased by ~ 25–30% for doubled saline thickness (nominally 100 μm) versus a decrease by ~ 20–25% for halved thickness (Musselman et al. 2023b). Additionally, our models assumed that A and B fibers were placed uniformly in all fascicles, but previous studies of pig cervical vagus nerve showed nerve vagotopy whereby fascicles are grouped in the nerve by physiological function and by afferents versus efferents (Aristovich et al. 2021; Blanz et al. 2023; Jayaprakash et al. 2022; Settell et al. 2020; Thompson et al. 2022). Incorporating fiber-type specific distributions within and/or across fascicles would increase the variance in thresholds for nerve responses due to increased variability in electrode-to-fiber distances. We also assumed discrete model A and B fiber diameters (i.e., 13 and 3 μm, respectively) for all individuals, but distributions of diameters and thus excitability likely exist within and across individuals, which would further increase inter-individual variability in nerve responses to stimulation.

Experimental data suggest that unidirectional propagation of action potentials might occur (Ahmed et al. 2020; Anholt et al. 2011), but across species and fiber types, our models exhibited bidirectional propagation. Although the experiments that we modeled did not report unidirectional propagation, it is possible that the fiber models that we used (McIntyre et al. 2002; Musselman et al. 2021; Tigerholm et al. 2014) lack representation of biophysics required to produce this phenomena.

## Conclusions

VNS produces variable nerve responses if stimulation parameters are not adjusted to account for differences in nerve morphology that occur within and across species. Applying uniform stimulation parameters within a species produced a large range of fiber-type specific responses, recycling stimulation parameters across species produced different patterns of nerve fiber activation between species, and linearly scaling stimulation parameters achieved the target response on average in the ending species but with a high degree of inter-individual variability. Systematic approaches for choosing stimulation parameters that account for individual- and species-specific differences are required to achieve equivalent nerve responses to VNS.

## Supplementary Information

Additional file 1.

## Data Availability

The datasets generated and/or analyzed during the current study are available in the Pennsieve repository: 10.26275/rpcu-wtfs.

## Abbreviations

CAP: Compound action potential
FEM: Finite element model
FMA-UE: Fugl-Meyer Assessment Upper Extremity scores
K: Ratio to scale stimulation currents
MRG: McIntyre-Richardson-Grill
VNS: Vagus nerve stimulation
