# Supplementary material for: Scaling of vagus nerve stimulation parameters does not achieve equivalent nerve responses across species
Source: Bioelectron Med. 2025 May 16;11:11. doi: 10.1186/s42234-025-00174-9 (PMC12083175; doi:10.1186/s42234-025-00174-9)
Supplement: Supplementary file 1 — Additional file 1. [file 42234_2025_174_MOESM1_ESM.docx]

# Appendix 1 – ReStore

The ReStore system consists of three major components: implanted pulse generator (IPG), external power-providing relay module, and external stimulation programming module (Sivaji et al., 2019). The IPG is wireless and is controlled by the programming module to deliver charge-balanced, biphasic current pulses of varying amplitudes, pulse widths, and frequencies. The IPG contains two platinum electrode contacts on the surface of a sealed rectangular prism encasing circuitry for stimulation and telemetry; the IPG is housed within a silicone casing that includes a “buckle” to attach the device to the nerve. The contacts align with cut-outs in the silicone casing, and they are thus recessed from inner surface of the housing that interfaces with the nerve. The geometry of the ReStore device is detailed in Supplemental Table 1 and Supplemental Figure 1.

Supplemental Table 1. Dimensions of the ReStore implantable stimulation device (S. Hayes, personal communication, December 6, 2022).

| **Diagram Label**  **(Supplemental Figure 1)** | **Part** | **Dimensions** | **Material** |
| --- | --- | --- | --- |
| A | Electrode contact spacing  (edge-to-edge) | 8 mm | Platinum iridium alloy |
| B | Electrode contact (length x width) | 2.5 mm x 4 mm | Platinum iridium alloy |
| C | IPG (length x width x height) | 13 mm x 8 mm x 3 mm | Glass |
| D | Opening for nerve in silicone casing | Approximately a semicircle with a radius of ~2.5-3 mm when placed before the 2^nd^ buckle. | Silicone |
| E | Silicone thickness | 1-1.5 mm | Silicone |
| F | Silicone cutout to expose the contacts | 4 mm x 4.5 mm | Silicone |
| G | Recess depth | 0.5 mm | Saline |


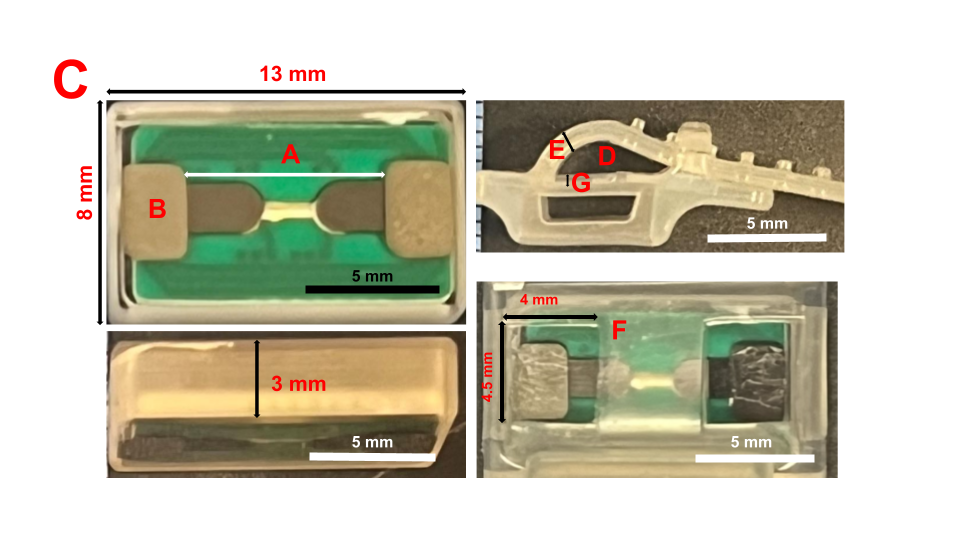


Supplemental Figure 1. ReStore system implantable stimulation IPG and silicone encasing.

In vivo studies with the ReStore system have been completed on the rabbit sciatic nerve, and a clinical trial of the system targeted at post-ischemic stroke rehabilitation is currently recruiting subjects. Supplemental Table 2 summarizes the design, stimulation parameters, and results of these studies.

Supplemental Table 2. Stimulation parameters and experimental data for three studies using the ReStore system.

| **Study Details** | **Stimulation Parameters** | **Validation** |
| --- | --- | --- |
| Male rabbit sciatic nerve, n = 4. Thresholds of sciatic nerve activation collected at time of stimulation. Sciatic nerve recruitment was determined by measuring the force of hindlimb muscle contraction in response to a range of stimulation amplitudes, and the threshold was determined by finding the lowest current amplitude that consistently caused any change in the force signal (Bucksot et al., 2019). | 0.5 s trains; amplitudes of 20-1600 µA; 100 µs/phase biphasic symmetric pulses at 10 Hz | mean ± SEM threshold of 351 ± 41.5 μA |
| Male rabbit sciatic nerve, n = 7. Thresholds of sciatic nerve activation collected at time of stimulation. Sciatic nerve recruitment was determined by measuring the force of hindlimb muscle contraction in response to a range of stimulation amplitudes, and nerve activation thresholds were established as the lowest stimulation amplitude required to induce a change in force greater than 3 times the standard deviation of the preceding 1 second of the baseline reading. Full fiber recruitment occurred when the force-amplitude curve plateaued (Sivaji et al., 2019). | 0.5 s trains; varying amplitudes; 100 µs/phase biphasic symmetric pulses at 10 Hz | Maximum fiber recruitment occurred at ~300 µA (see Figure 8 of (Sivaji et al., 2019). |
| Human vagus nerve, n = 30. Adverse effects to be recorded from week 1 through study follow-up two years after final stimulation session (NCT04534556). | 0.5 s trains; 0.8 mA; 100 µs/phase biphasic symmetric pulses at 30 Hz | N/A, study not yet published |

# Appendix 2 – SetPoint

The SetPoint Medical VNS system is a wirelessly controlled implantable pulse generator (IPG) with integrated leads (“MicroRegulator”) and a flexible silicone encasing (“POD”); the POD holds the MicroRegulator flush to the nerve. The device is charged wirelessly by an external module and stimulation parameters are controlled via telemetry (Genovese et al., 2020). The device has an elliptical opening for the nerve available in 3 sizes, with a major axis of 2, 3, or 4 mm (Faltys et al., 2013). The MicroRegulator contains semi-elliptical bipolar electrodes (spaced 8 mm apart, center-to-center) made of a platinum-iridium alloy. The geometry of the SetPoint device is detailed in Supplemental Table 3 and Supplemental Figure 2.

Supplemental Table 3. Dimensions of the SetPoint implantable stimulation device (Faltys et al., 2013).

| **Diagram Label (Supplemental Figure 2)** | **Part** | **Dimensions** | **Material** |
| --- | --- | --- | --- |
| A | Electrode contact spacing  (center-to-center) | 8 mm | Platinum iridium alloy |
| B | Electrode contact length | 5 mm | Platinum iridium alloy |
| C | Cuff length | 20 mm | Silicone |
| D | Elliptical opening | 3 sizes available, defined by major axis x minor axis: 4 mm x 3 mm, 3 mm x 2.25 mm, 2 mm x 1.5 mm | Silicone |
| E | Cuff wall thickness | 0.2 mm | Silicone |


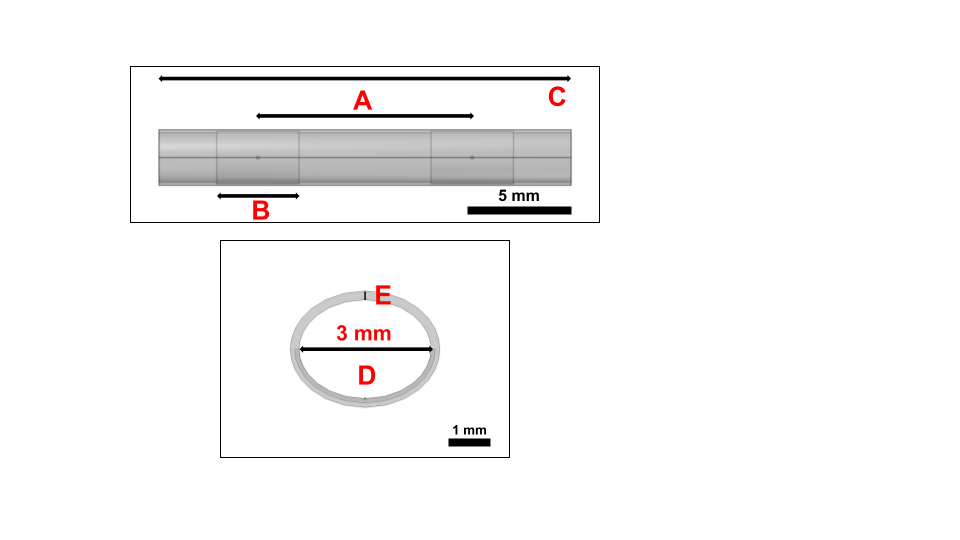


Supplemental Figure 2. Diagram of the SetPoint implantable stimulation device, shown for the 3 mm x 2.25 mm opening size (Faltys et al., 2013).

The SetPoint device delivers biphasic symmetric rectangular pulses (250 μs/phase) at 10 Hz. The device can deliver at up to 2.5 mA in increments of 0.1 mA though in the primary clinical trial (Genovese et al., 2020), current levels were titrated based on patient tolerance to a maximum of 2.5 mA. The mean current amplitude delivered across 11 patients was 1.52 mA.

# Appendix 3 – Nerve Deformation for ReStore and SetPoint

## ReStore Nerve Deformation and Cuff Choice/Orientation

We modeled the 2 and 3 mm ReStore cuffs (Appendix 1 – ReStore) based on the cuff’s two most used buckle closure locations: “before 2^nd^ buckle” results in a 3 mm-high cuff opening and “before 1^st^ buckle” results in a 2 mm-high cuff opening.


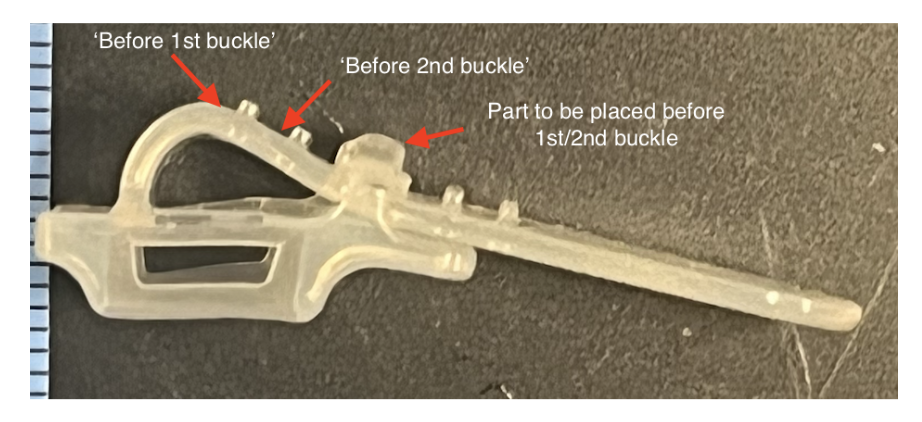


Supplemental Figure 3. Image of the ReStore cuff electrode showing the buckles used to secure the cuff on the nerve. The tick marks on the left edge are spaced by 1 mm.

The ReStore cuff has an opening that is approximately semi-circular. Degree of cuff rotation on the nerve was determined by aligning the minor (vertical) axis of the nerve with the vertical axis of the cuff’s semi-circular opening. We used the major and minor axes of the best-fit ellipse of each nerve cross section to determine if nerve deformation was required for it to fit in the ReStore cuff. If the best-fit ellipse of the nerve had a minor axis greater than 2.3 mm, we used the 3 mm cuff size, and the sample cross section was deformed to an ellipse with a minor axis of 2.3 mm while preserving cross-sectional area. The minimum minor axis size of 2.3 mm was used as a threshold for deformation within the 3 mm cuff to account for 0.5 mm of surrounding encapsulation tissue and 0.2 mm of surrounding saline fill (2.3 + 2*0.25 + 2*0.1 = 2.3 + 0.5 + 0.2 = 3 mm).

If the minor axis of the best-fit ellipse was between 2 and 2.3 mm, we used the 3 mm cuff size, and no nerve deformation was necessary.

If the minor axis of the best-fit ellipse was between 1.3 and 2 mm, we used the 2 mm cuff size, and the sample cross section was deformed to an ellipse with a minor axis of 1.3 mm while preserving cross-sectional area. As described above for the 3 mm cuff, the minimum minor axis size of 1.3 mm was used as a threshold for deformation within the 2 mm cuff to account for 0.5 mm of surrounding encapsulation tissue and 0.2 mm of surrounding saline fill (1.3 + 2*0.25 + 2*0.1 = 1.3 + 0.5 + 0.2 = 2 mm).

Finally, if the minor axis of the best-fit ellipse was less than 1.3 mm, we used the 2 mm cuff size and no nerve deformation was necessary.

## SetPoint Nerve Deformation and Cuff Choice/Orientation Procedure

The SetPoint cuff opening is elliptical (4:3 major-to-minor axis ratio) and is available in three major axis sizes: 2, 3, and 4 mm. We determined the appropriate cuff size for each nerve based on cross-sectional areas of the cuffs and the nerve. First, we selected the smallest cuff diameter for which the cross-sectional area of the cuff minus cross-sectional area for a 10 μm saline layer along the inner cuff boundary was larger than the nerve cross-sectional area (Supplemental Table 4). All models included a 0.1 mm saline layer and 0.25 mm encapsulation tissue between the cuff and the nerve (similar to ReStore cuff modeling), but cuff size was chosen based on a 10 μm saline layer minimum. For example, if a nerve sample has a cross-sectional area of 4.34 mm^2^, it would be assigned to the 3 mm cuff, which has nearest-higher cross-sectional area of 5.21 mm^2^. If there was not enough space to accommodate both the complete saline and encapsulation tissue layers, the body of the nerve would take priority, displacing any excess encapsulation tissue or saline as needed to fit within the cuff. However, a minimum 10 µm saline layer surrounding the nerve inside the cuff was preserved. Because encapsulation tissue directly surrounds the nerve, with the saline layer forming an outer shell, any space constraints resulted in the encapsulation tissue being overridden prior to the saline layer. This constraint ensured that at least a 10 µm saline layer was always maintained.

Supplemental Table 4. SetPoint cuff dimensions in relation to nerve sample geometry.

| **Major axis of cuff opening (mm)** | **Minor axis of cuff opening (mm)** | **Cross-sectional area of cuff opening (mm^2^)** | **Cross-sectional area of 10 μm saline layer (mm^2^)** | **Cross-sectional area of cuff opening minus cross-sectional area of saline layer (mm^2^)** | **Range of nerve cross-sectional areas (mm^2^)** |
| --- | --- | --- | --- | --- | --- |
| 2 | 1.5 | 2.36 | 0.6 | 2.30 | 0 – 2.30 |
| 3 | 2.25 | 5.30 | 0.9 | 5.21 | 2.31 – 5.21 |
| 4 | 3 | 9.42 | 0.11 | 9.31 | 5.22 – 9.31 |

Cuff rotation was determined by aligning the minor axes of the nerve with the minor axis of the cuff’s elliptical opening. We determined whether a given nerve required deformation to fit within the selected cuff, based on the major and minor axes of the nerve’s best-fit ellipse. If both the major and minor axes of the nerve were smaller than the chosen cuff dimensions, no nerve deformation was performed. If the nerve’s major axis was larger than the cuff’s major axis, the nerve was deformed to an ellipse while preserving its cross-sectional area using the cuff’s major axis. In this case, the nerve’s major axis was deformed and decreased to precisely the length of the cuff’s major axis (minus saline thickness), and based on this deformation, the nerve’s minor axis length would be calculated and increased while preserving cross-sectional area. Similarly, if the nerve’s minor axis was larger than the cuff’s minor axis, the nerve was deformed to an ellipse while maintaining the cross-sectional area using the cuff’s minor axis. Here, the nerve’s minor axis was decreased to the length of the cuff’s minor axis (minus saline thickness), and based on this deformation, the nerve’s major axis was calculated and increased to preserve nerve cross-sectional area.

# Appendix 4 – Finite Element Models of Rat VNS for Stroke, Inflammation, and Heart Failure

We modeled published rat VNS studies, using their described cuff dimensions as described below (Supplemental Figure 4).

### Rat VNS for Stroke (Porter et al., 2012)

“Two Teflon-coated multistranded platinum iridium (0.006”) wires were connected to a 4 mm section of Micro-Renethane tubing (1.8 mm inner diameter). The wires were spaced 2 mm apart along the length of the tubing. An 8 mm region of the wires lining the inside circumference of the tube was stripped of the insulation. A cut was made lengthwise along the tubing to allow the cuff to be wrapped around the nerve and then closed with silk threads. This configuration resulted in the exposed wires being wrapped around the vagus nerve at points separated by 2 mm, while the leads exiting the cuff remained insulated.”

### Rat VNS for Inflammation (Kin et al., 2021)

“We used cuff-type electrodes purchased from Unique Medical Co., Ltd. (Tokyo, Japan) (Figure 2a). Electrodes were composed of two curved silver wires (0.08 mm diameter) covered with a 4 mm section of polyethylene tubing (outer/inner diameter: 1.0 mm/0.5 mm). The silver wires were aligned 1.5 mm apart in parallel inside the cuff. A cut was made lengthwise along the tubing to allow the cuff to be wrapped around the nerve and then closed by a suture (Figure 2b). The insulation was removed to provide conductivity, allowing bipolar stimulation limited in surrounding the nerve. These electrodes were designed based on a similar method used by several previous studies [16–18].”

### Rat VNS for Heart Failure (Li et al., 2019)

“To increase the flexibility and endurance of the lead, we designed a coil structure using a thin stainless wire (OD 0.03 mm, coated with polyurethane; Unique Medical Co., Ltd., Tokyo, Japan). A segment (4 mm) of a commercially available silicone tube (OD 1 mm, ID 0.5 mm) was used as a cuff around the nerve. A longitudinal slit was made on the tube, through which the nerve was placed. The wires were penetrated through the thin wall and bent against the cuff, parallel to the cuff’s long axis.”


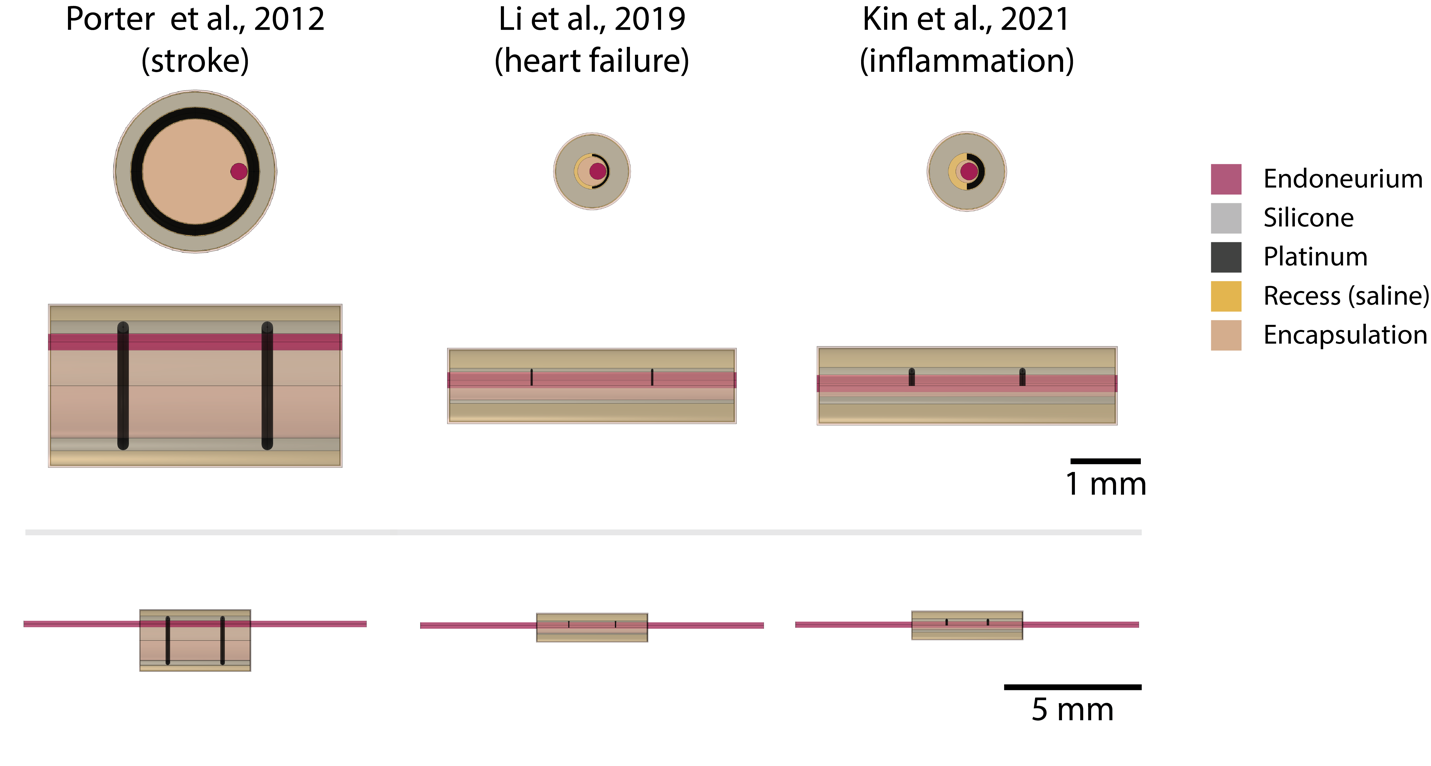


Supplemental Figure 4. Finite element models of three cuff electrodes (columns) used in published studies of rat VNS. The images in the top two rows share the same scale bar (1 mm), and the images in the bottom row share the same scale bar (5 mm).

# Appendix 5 – Concomitant Fiber Responses with Activation of 50% of A Fibers


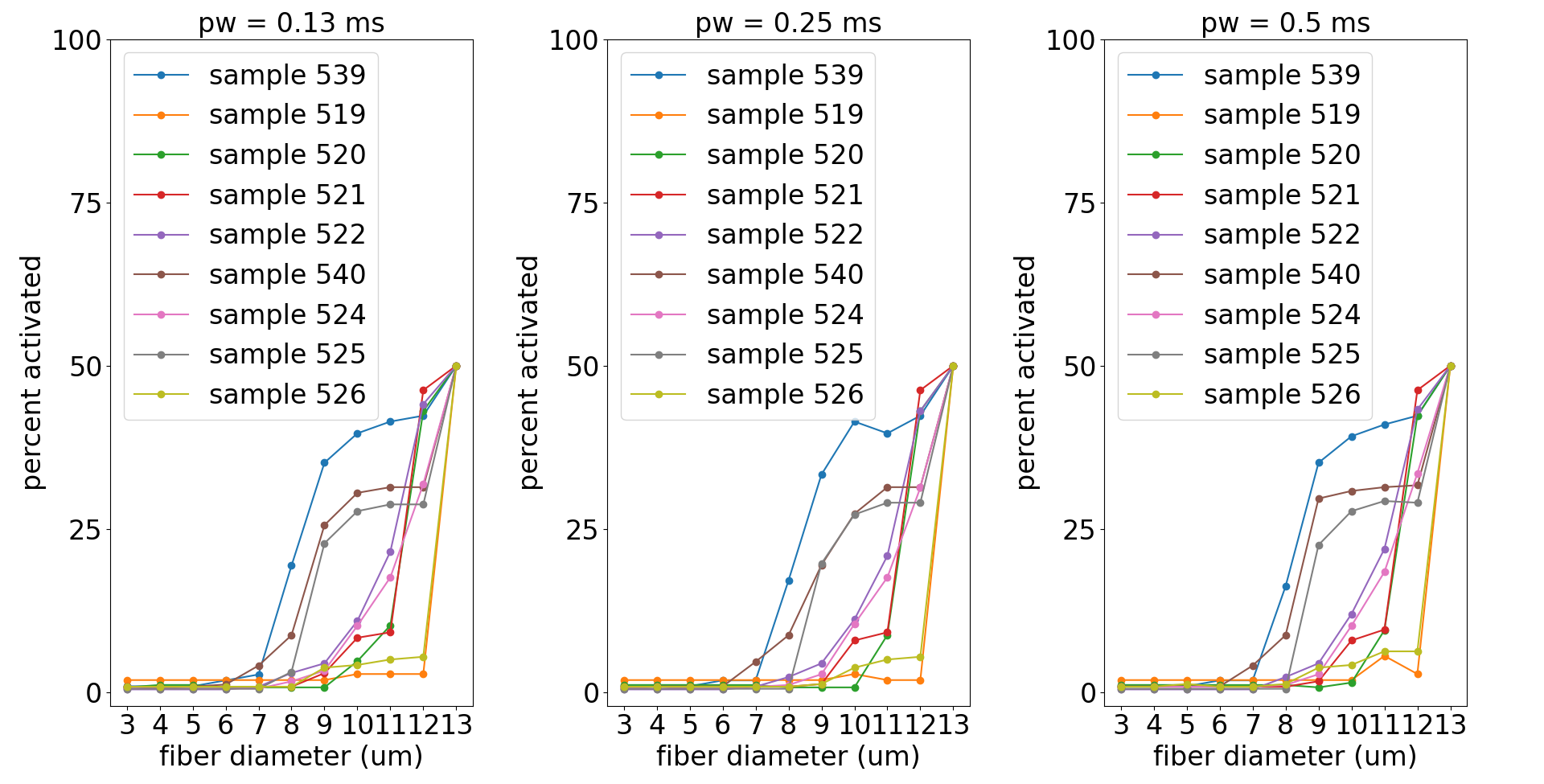


Supplemental Figure 5. Fiber activation responses to I_50,A_ in humans, where A fibers were modeled as 13 µm in diameter. We delivered the I_50,A_ for each individual and calculated the percent of fibers (by diameter) that were above or below threshold. Concomitant activation of smaller fiber diameters varied across individuals. Each panel is for a biphasic rectangular pulse with a different pulse width.


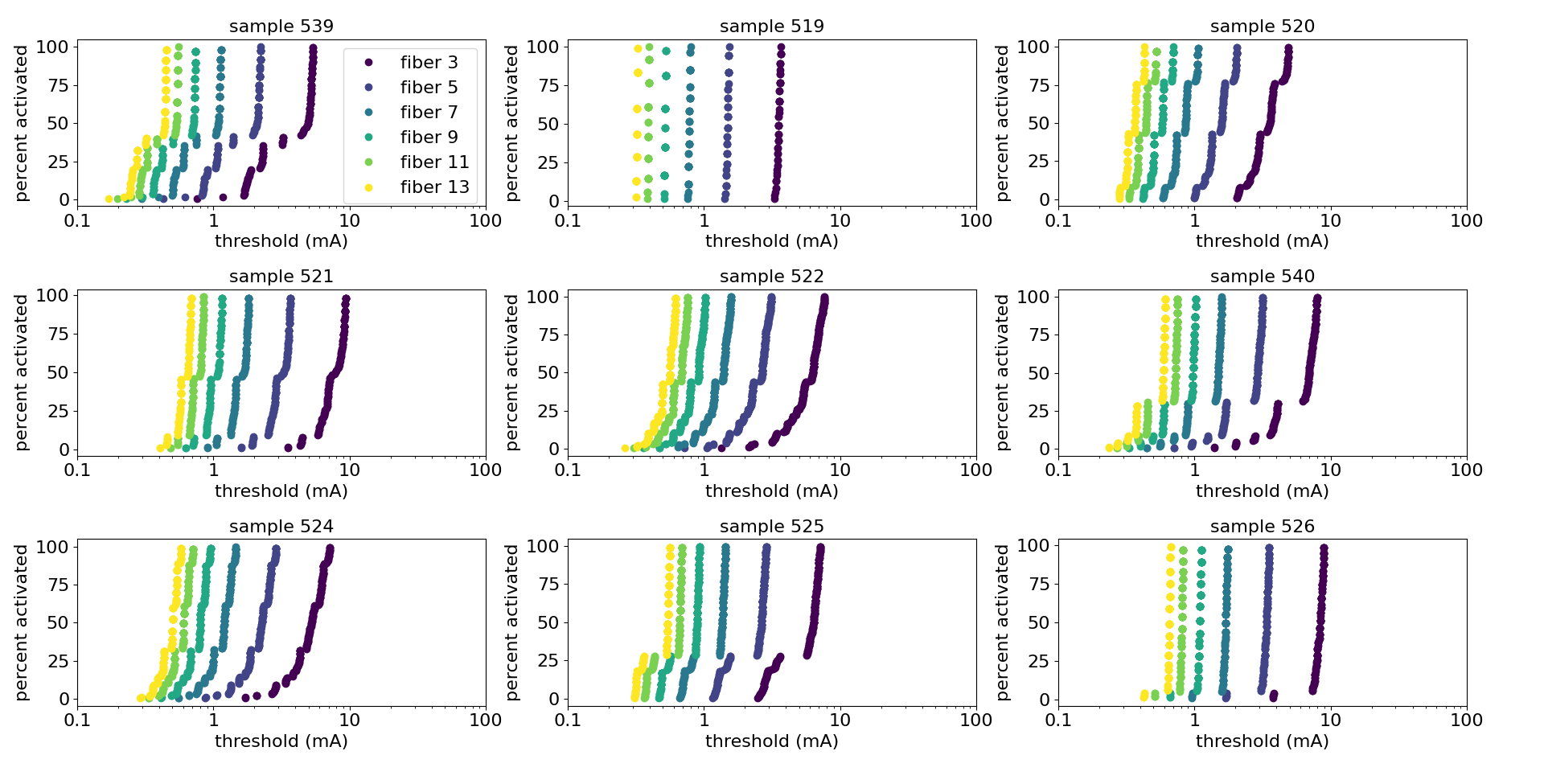


Supplemental Figure 6. Dose-response curves by fiber diameter for each human nerve. The data shown are for a 0.25 ms/phase biphasic pulse. Fascicle-based recruitment produces a stepped-response.

# Appendix 6 – Histograms of Linear Scaling Factors for Additional Pulse Widths


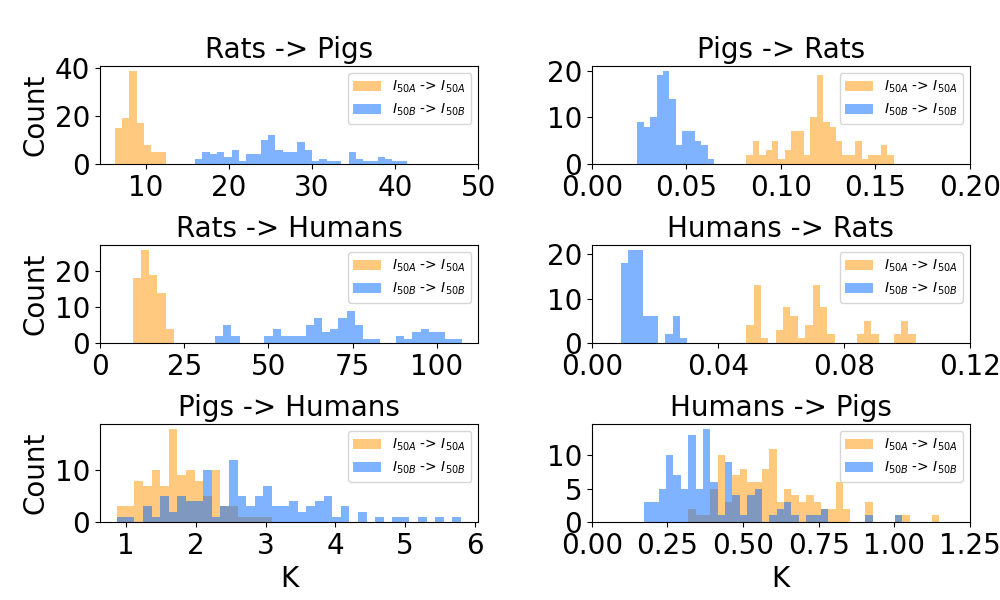


Supplemental Figure 7. Histograms of linear scaling factors (K) between stimulation amplitudes that activate 50% of nerve fibers (i.e., I_50_ for A or B fibers) between all pairs of all individuals of different species with 0.13 ms pulse width; data for pulse width of 0.25 ms are provided in Figure 4. The left and right columns provide the Ks from smaller to larger species and from larger to smaller species, respectively.


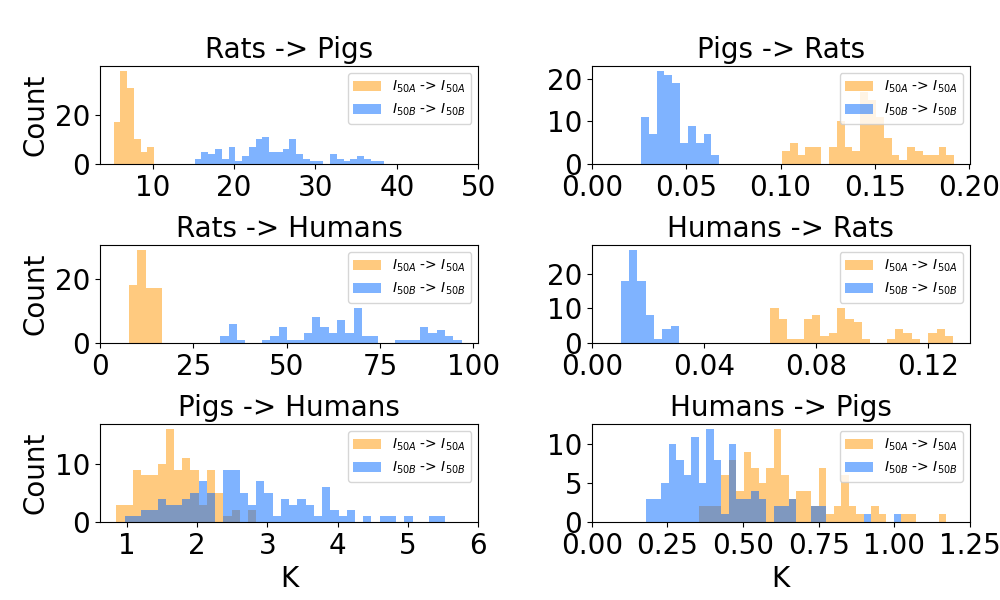


Supplemental Figure 8. Histograms of linear scaling factors (K) between stimulation amplitudes that activate 50% of nerve fibers (i.e., I_50_ for A or B fibers) between all pairs of all individuals of different species with 0.25 ms pulse width. The left and right columns provide the Ks from smaller to larger species and from larger to smaller species, respectively.


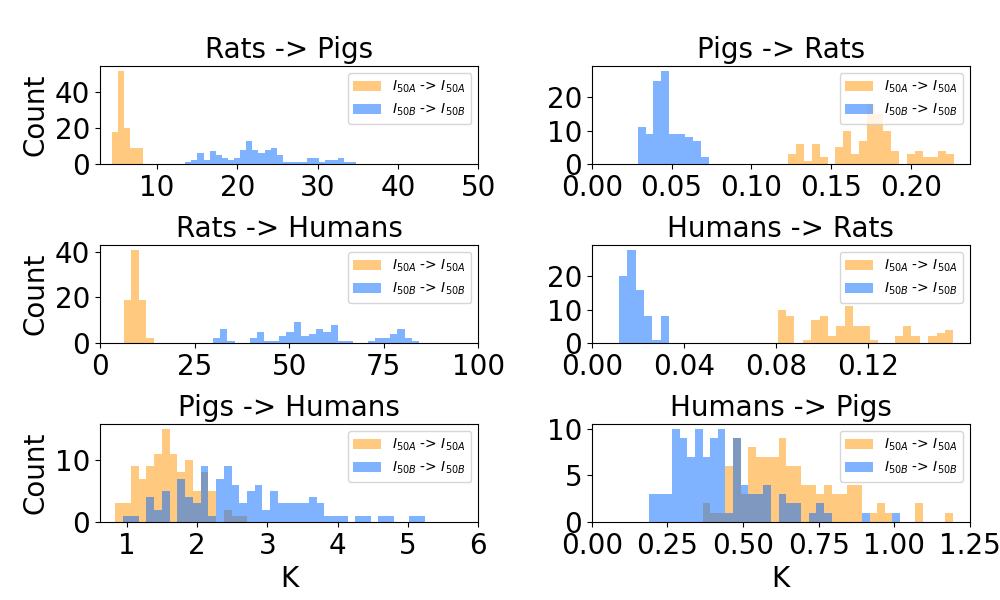


Supplemental Figure 9. Histograms of linear scaling factors (K) between stimulation amplitudes that activate 50% of nerve fibers (i.e., I_50_ for A or B fibers) between all pairs of all individuals of different species with 0.5 ms pulse width; data for pulse width of 0.25 ms are provided in Figure 4. The left and right columns provide the Ks from smaller to larger species and from larger to smaller species, respectively.
